# Supplementary material for: Divergent Deborah number-dependent transition from homogeneity to heterogeneity
Source: Nat Commun. 2023 Sep 26;14:6003. doi: 10.1038/s41467-023-41738-0 (PMC10522598; doi:10.1038/s41467-023-41738-0)
Supplement: Supplementary file 1 — Supplementary Information [file 41467_2023_41738_MOESM1_ESM.pdf]

Supplementary Information

Divergent Deborah number-dependent transition from homogeneity to heterogeneity

*Dan Xu[1], Yang Yang[1], Lukas Emmerich[2], Yong Wang[3], Kai Zhang[1]**

Address

1 Sustainable Materials and Chemistry, Department of Wood Technology and Wood-based Composites, University of Göttingen, Büsgenweg 4, D-37077 Göttingen, Germany.

2 Department of Wood Biology and Wood Products, University of Göttingen, Büsgenweg 4, D-37077 Göttingen, Germany.

D-37077 Göttingen, Germany.

3 Laboratory for Fluid Physics, Pattern Formation and Biocomplexity, Max Planck Institute for Dynamics and Self-Organization, Am Faßberg 17, D-37077 Göttingen, Germany.

*Corresponding author. E-mail: kai.zhang@uni-goettingen.de

This file includes:

Supplementary Note 1

Supplementary Fig 1-39

Supplementary Table 1-3

Supplementary References

Supplementary Note 1 - The volatility evaluation of various media

The liquid phase velocity is small compared to the moist air velocity, which means advective transport rate (in the inner part of hydrogel) is lower than diffusive transport rate (on the interface of hydrogel/air). Therefore, the water velocity in hydrogel is determined by gas phase pressure gradient (based on Dancy' law). Under the assumption that all hydrogels are of similar porous structures, the whole evaporation process is mainly dominated by the volatility of media.

Here, three media were applied in the system, including the original sodium hydroxide, borax-NaOH buffer (pH = 10.00) (buffer), a faster evaporation system containing 10% ethanol and a slower evaporation system containing 5% glucose. To quantitatively describe the volatility difference between these media, an approximate calculation was applied based on Henry's law and Raoult's low, and the saturated vapor pressure was set as the evaluation criterion. Under constant ambient pressure and temperature, the volatility and the saturated vapor pressure is positive correlation, which means that the liquid of high saturated vapor pressure allows for easier transition from liquid to gas. Here, the temperature was set as 293 K and the ambient pressure was 1 atm.

To simplify things, we would use the saturated vapor pressure of water to refer to that of buffer solution. Thus, the saturated vapor pressure of the system is around 2.34 kPa.

As for the faster evaporation system containing 10% ethanol, the Henry's law was applied.

$$P = P_{water} + P_{ethanol} = x_{water}P^0_{water} + x_{ethanol}P^0_{ethanol} \tag{1}$$

Here, x refers to the molar percentage of chemicals, and the P^0 means the saturated vapor pressure of chemicals. Herein, the saturated vapor pressure of the medium is 2.69 kPa.

As for the slower evaporation system containing 5% glucose, the Raoult's law was applied.

$$P = P^0_{water} - \Delta P = x_{water}P^0_{water} \tag{2}$$

Here, ΔP is the decreased vapor pressure of medium due to colligative properties of glucose solution. Thus, the saturated vapor pressure of the medium is 2.22 kPa.

In brief, the order of evaporated speed can be regarded as:

10% ethanol > buffer > 5% glucose

The experimental data are also consistent with the above conclusion. Typically, the free liquid drops were placed on the glass slide. The evaporation area is kept constant (Constant contact radius mode). The degree of air drying completion was defined as evaporated mass/original mass of the drops. When the value reaches 1, it means that the evaporation process is complete.

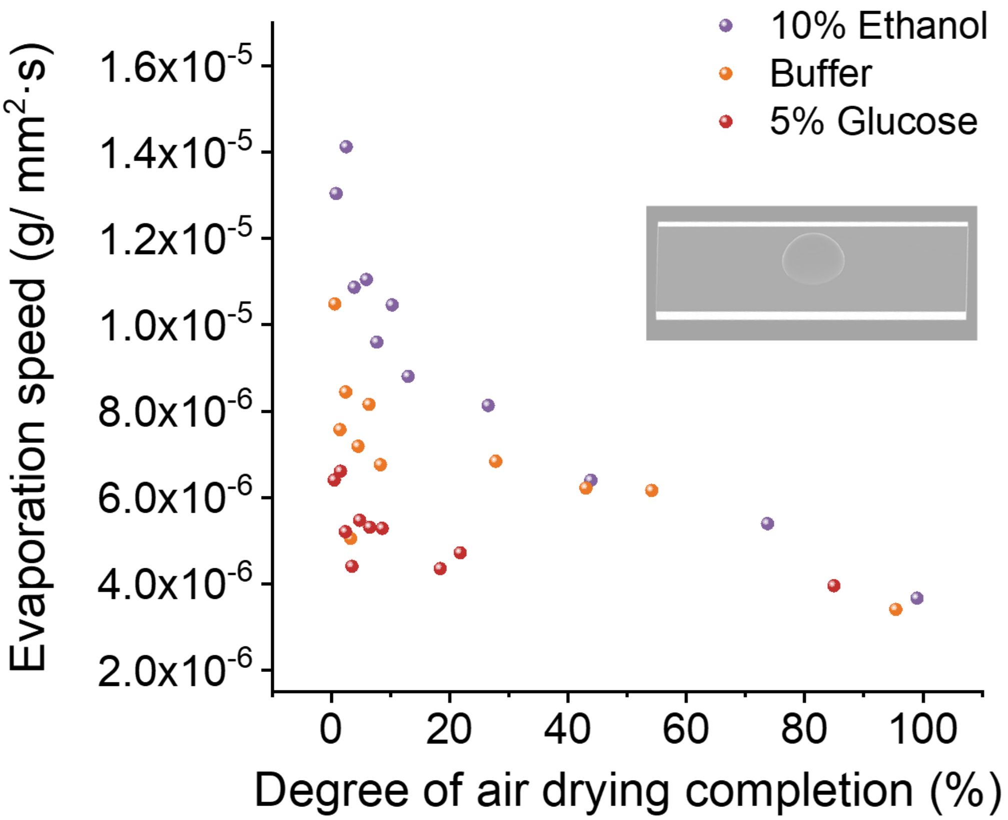

Supplementary Fig. 1. The evaporation speeds of different media in air drying process.

From the above experimental data, it is clear that the difference in evaporation rate between these three media follows the theoretical expectation.

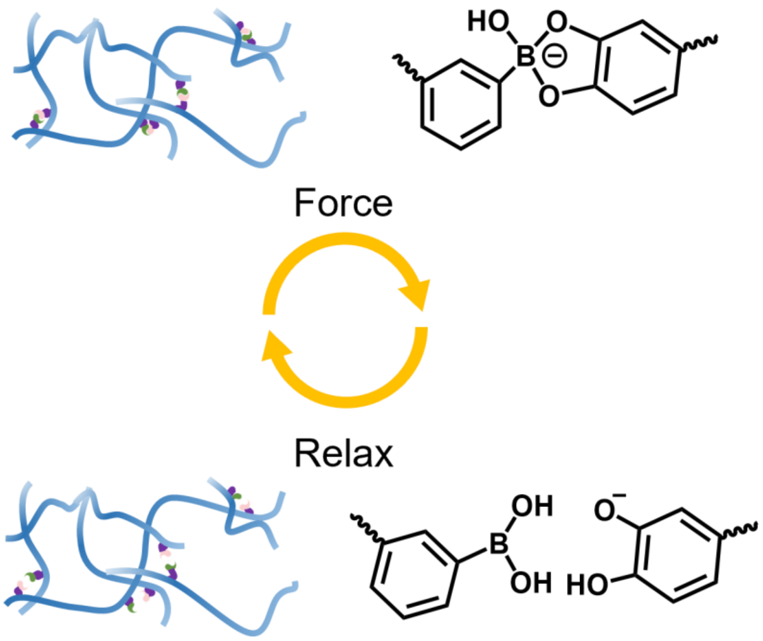

Supplementary Fig. 2. The energy dissipation mechanism of dynamic hydrogel in the process of uniaxial stretching via the reconstruction of non-covalent bonding.

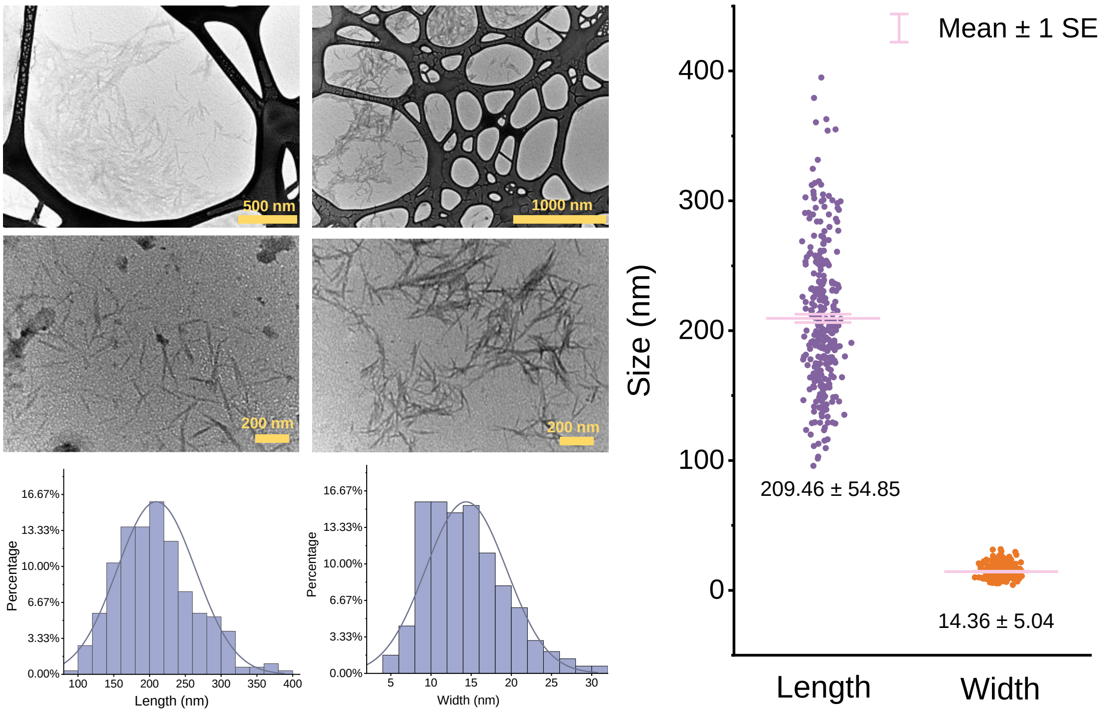

Supplementary Fig. 3. Typical TEM images of TEMPO-CNCs. The length and width were analyzed, and the number of samples for statistical analysis was 300.

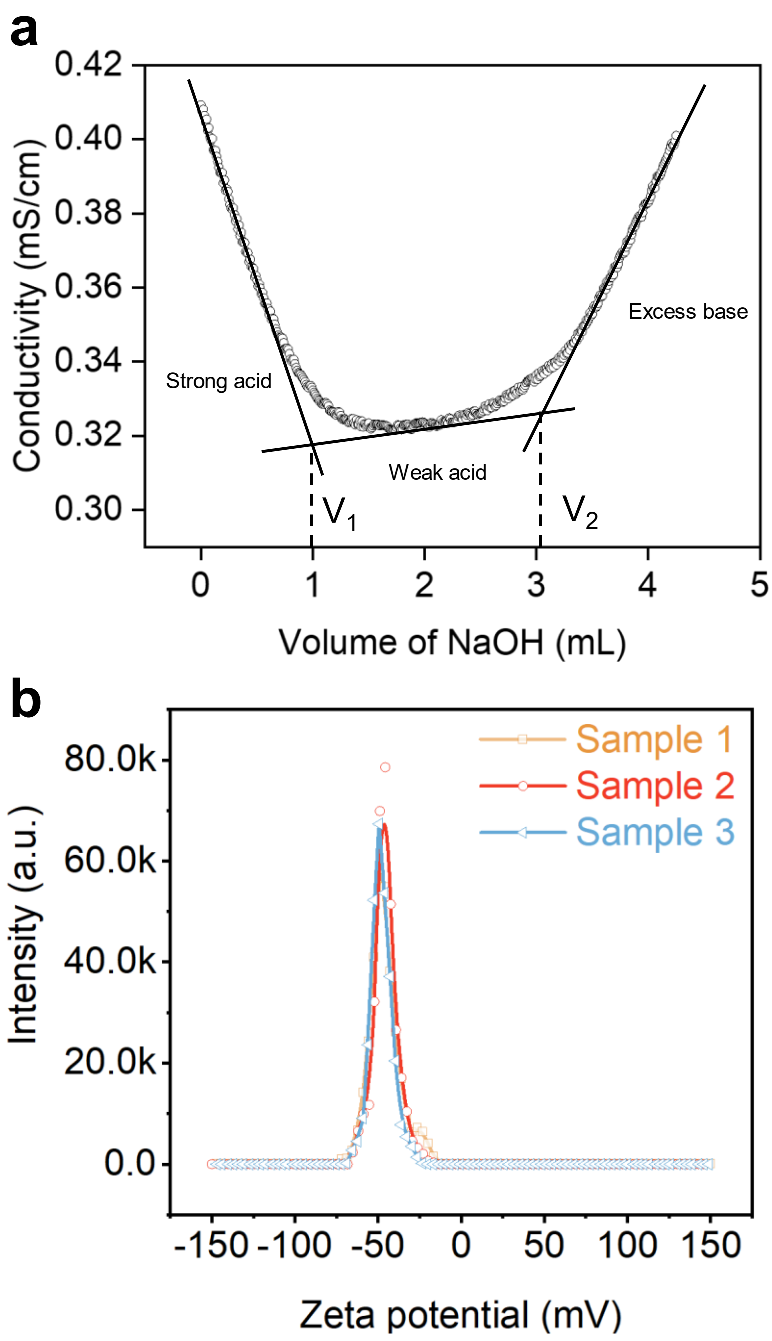

Supplementary Fig. 4. The physical properties of prepared CNCs. **a**) Typical conductometric titration curve of CNCs; **b**) Zeta potential distribution of CNCs. (N = 3)

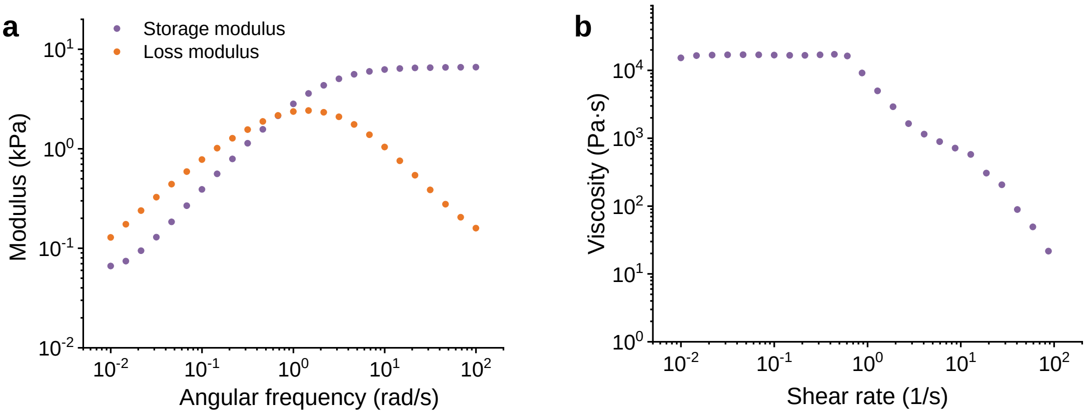

Supplementary Fig. 5. The rheological properties of dynamic crosslinked hydrogel. a) Frequency sweep; **b**) Shear thinning properties.

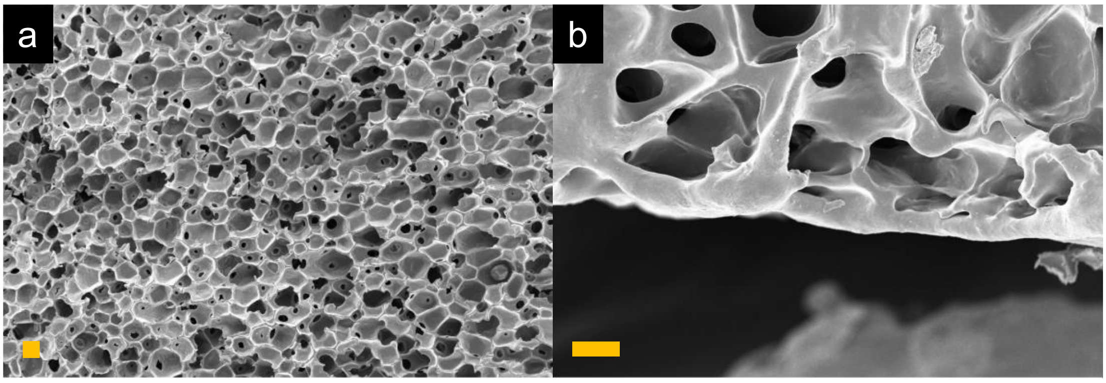

Supplementary Fig. 6. SEM images of virgin CNC hybrid hydrogel (without stretching and air-drying) after freeze-drying. a) inner part; **b**) outer part. Scale bar: 10 µm.

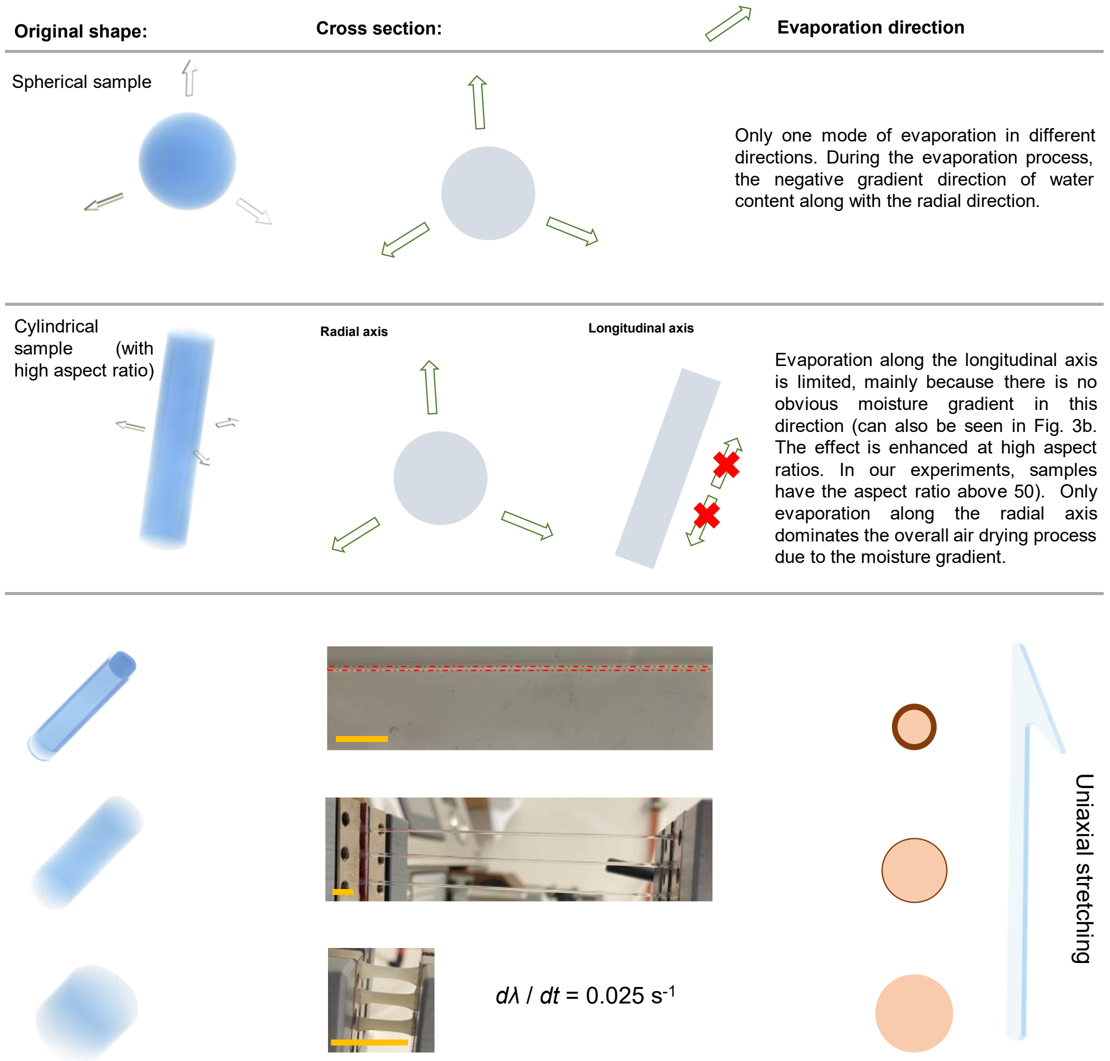

Supplementary Fig. 7. Schema of plants inspired spontaneously formed core-sheath structural hybrid fibers. Based on numerical analysis, it has been demonstrated that the air drying process is isotropic for cylindrical samples. The initial strain rate was defined as $d\lambda$ / dt to eliminate the scale effect. Scale bar: 5 mm

The diffusion of water in samples was simplified and described using Fick's Low. Thus, the evolution of water concentration depends on the position (x) and diffusion time (t). The concentration of water with respect to time was described at:

$$\frac{\partial \varphi}{\partial t} = D\frac{\partial^2 \varphi}{\partial x^2} \tag{3}$$

Where φ is the concentration and D is the diffusion coefficient. Thus, for the spherical sample, the diffusion process was the same along the evaporation direction. When the sample changes to cylindrical specimens, there are two possible diffusion pathways, one along the radial direction and the other one along the longitudinal direction. Taking

the central zone of cylinder samples, we can define the speed of concentration change in radial direction (v_r) as Eq. (4) and in longitudinal direction (v_l) as Eq. (5)

$$v_r = \frac{\partial \varphi_r}{\partial t} = D\frac{\partial^2 \varphi}{\partial r^2} \tag{4}$$

$$v_l = \frac{\partial \varphi_l}{\partial t} = D\frac{\partial^2 \varphi}{\partial (l/2)^2} \tag{5}$$

We can see that the ratio between these two independent directions is proportional to the square of aspect ratio (cylinder). For example, for the cylinder sample with a high aspect ratio, such as more than 50, the v_r is 2500 times v_l. Therefore, for high aspect ratio cylindrical samples, the diffusion along the radial direction dominates the overall water diffusion process.

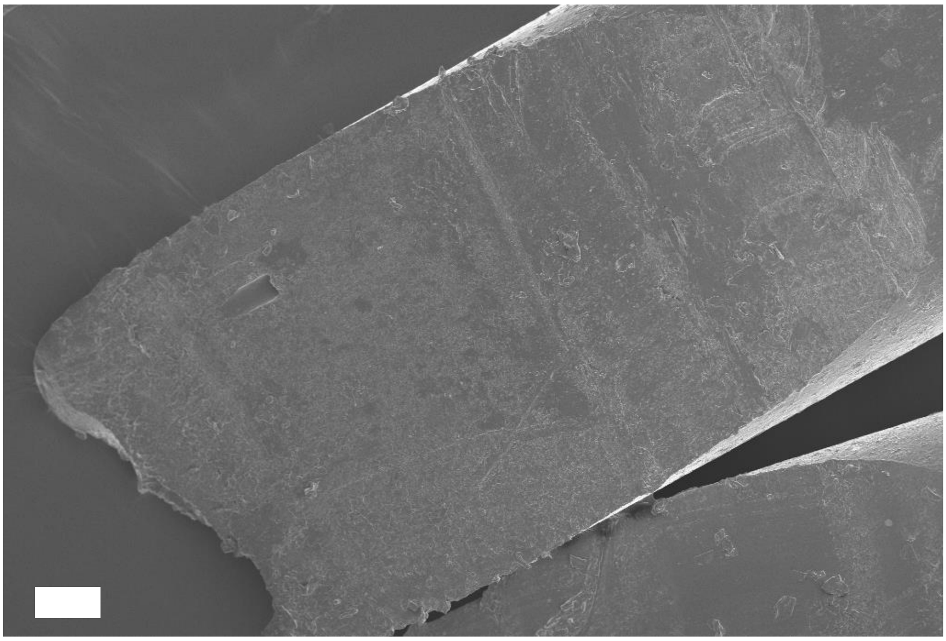

Supplementary Fig. 8. SEM image of the bevel section in the xerogel fiber. Scale bar:100 μm.

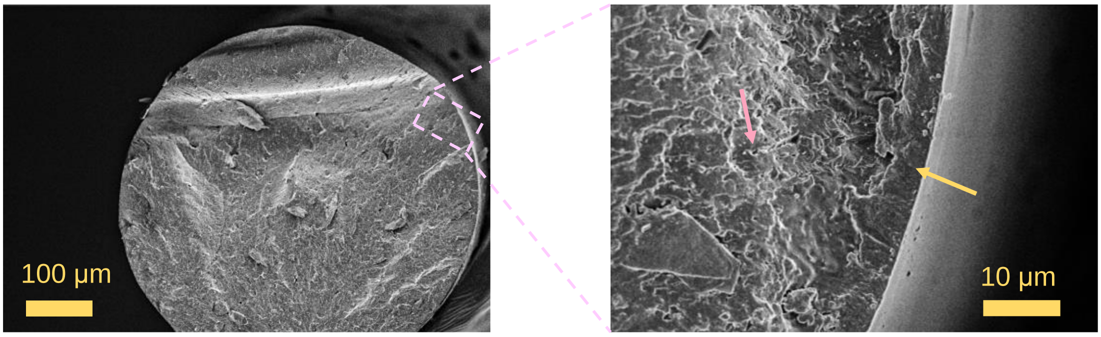

Supplementary Fig. 9. SEM image of the cross section of a xerogel fiber. The colored arrows indicate different regions of different structural features.

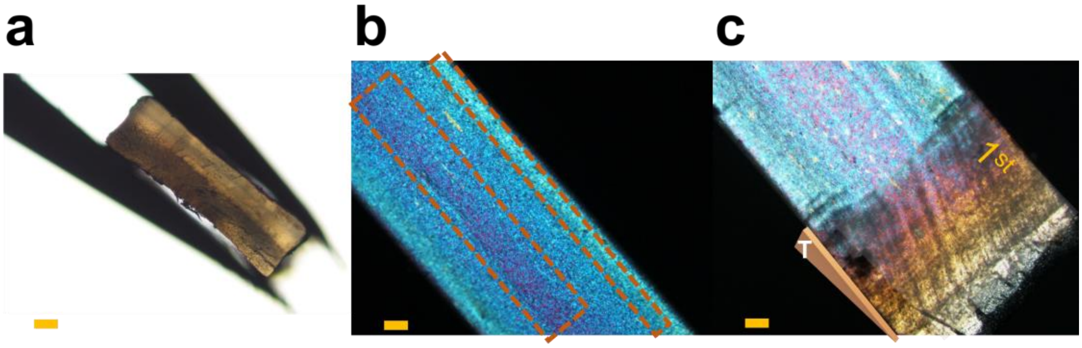

Supplementary Fig. 10. Optical and POM images of CNC hybrid xerogel films. **a**) Optical images of xerogel film cross section; and the POM images of bevel xerogel fibers, including **b**) Surface and **c**) Bevel section. Scale bar: 100 μm.

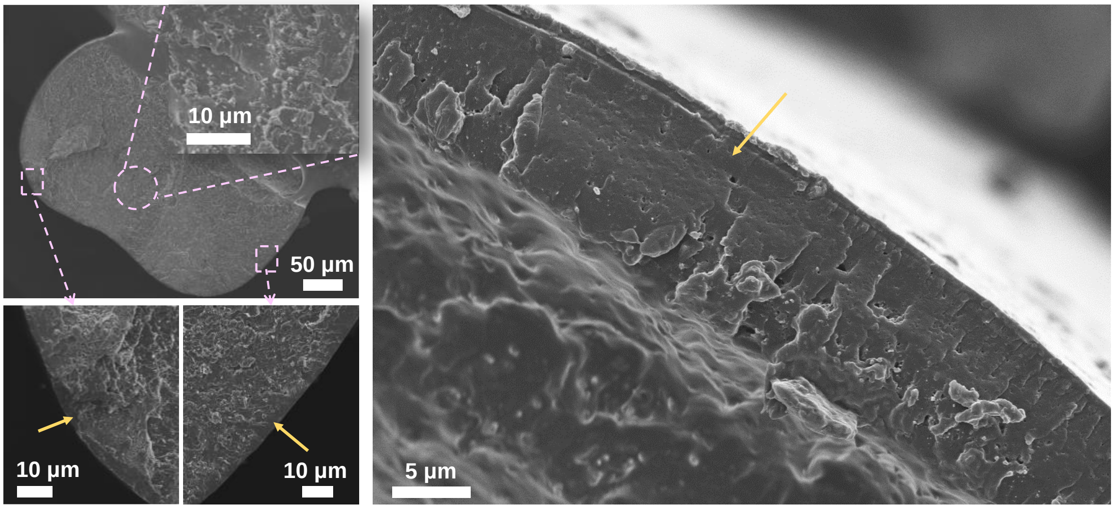

Supplementary Fig. 11. The microstructures of xerogel fibers with rectangular cross section. The heterogeneous structures were distributed around the boundary zone. In general, the inner part was rough with cracks while the outer part was relatively smooth.

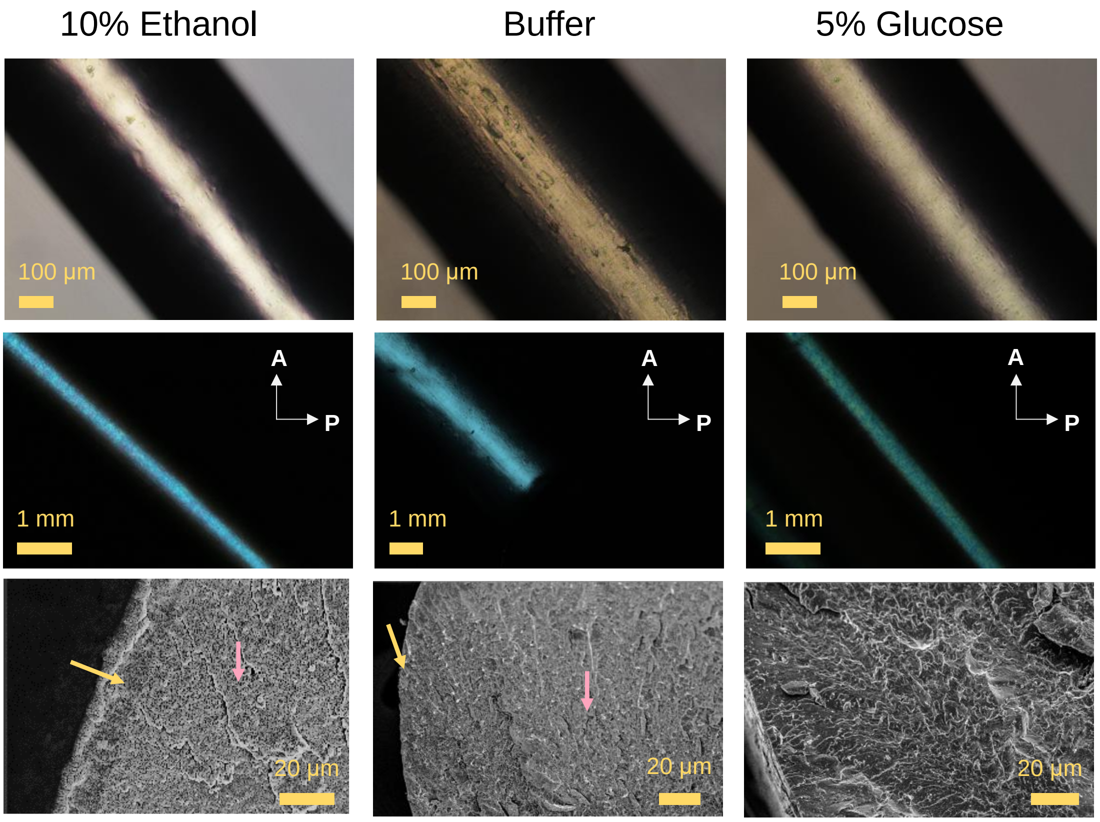

Supplementary Fig. 12. The optical microscopy images, polarized microscopy images and SEM images of various obtained xerogel fibers. The colored arrows indicate the regions with different structural features.

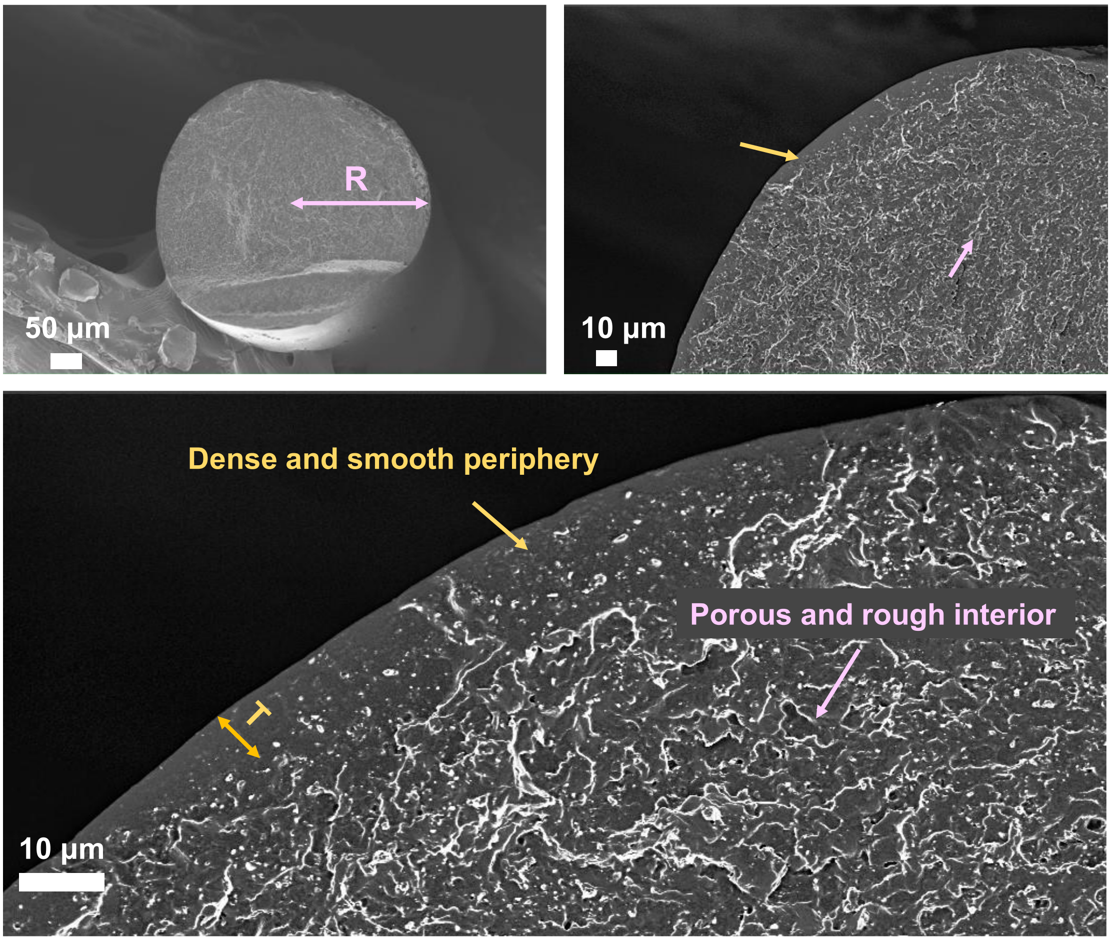

Supplementary Fig. 13. The microstructures of xerogel fibers with circular cross section. T: the thickness of outer part; R: the radius of xerogel samples.

Supplementary Table 1. The physics parameters used to simulate the air drying of hydrogel fibers

293.15 [K]	Ambient temperature
0.05 [m/s]	Freestream velocity
0.3	Ambient relative humidity
0.3	Irreductible liquid phase saturation
0.59	Porosity
0.98*1e-12 [m^2]	Permeability
0.8 [W/(m*K)]	Porous matrix thermal conductivity
6500 [J/(kg*K)]	Porous matrix heat capacity
1200 [kg/m^3]	Porous matrix density

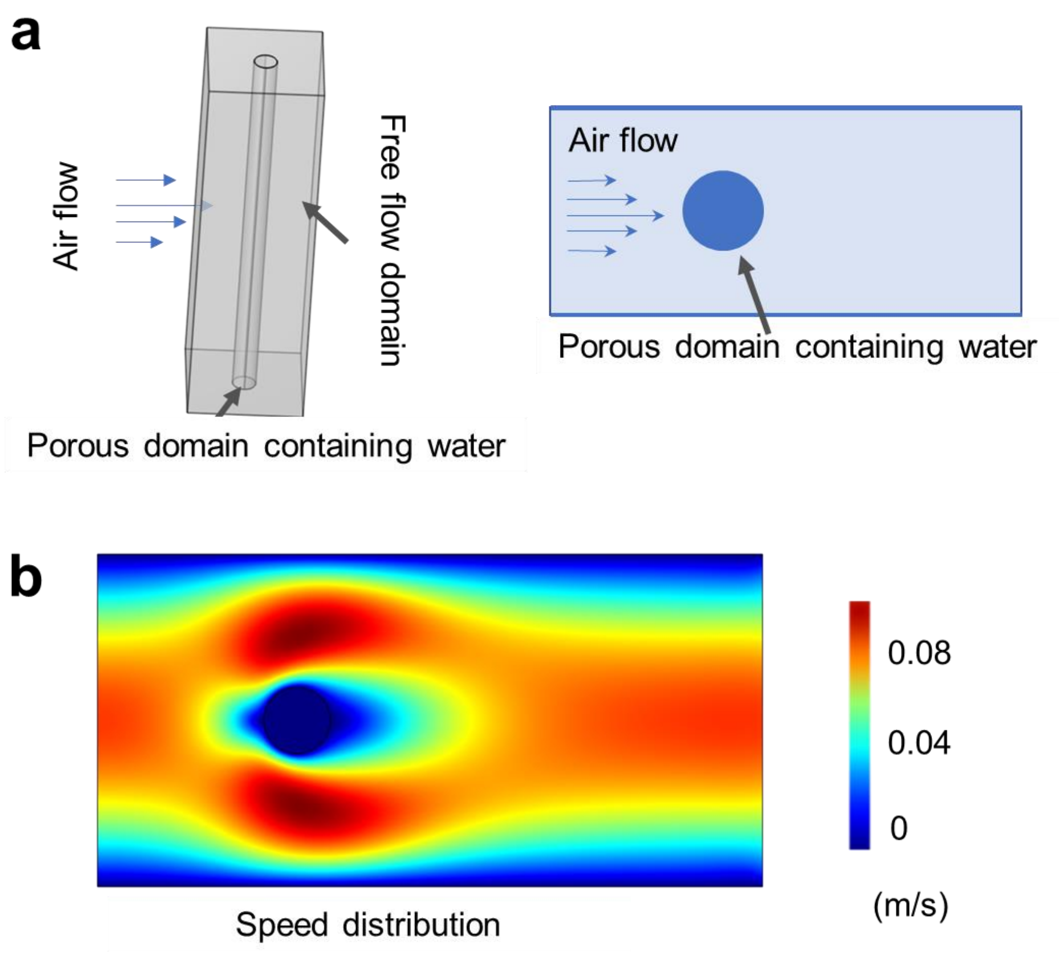

Supplementary Fig. 14. The physical model to simulate the evaporation process for hydrogels. a) A full-scale model was used to simulate the drying process of hydrogel fiber in the stage of air drying. **b)** The wind speed distribution near the surface of hydrogel fiber.

The Reynolds number (*Re*) of this system could be approximately calculated as below

$$Re = \rho v \frac{d}{\mu} \tag{6}$$

Here, the ρ is the density of air (~1.2 kg/m^3), v is the flow speed (set as 0.05 m/s), d is the characteristic linear dimension (for cylinders is the diameter, ~0.001 m) and μ is the dynamic viscosity of air (~1.85×10^{-5} Pa·s). Thus the *Re* in our system is around 0.3, revealing the flow of air is the stable laminar flow.

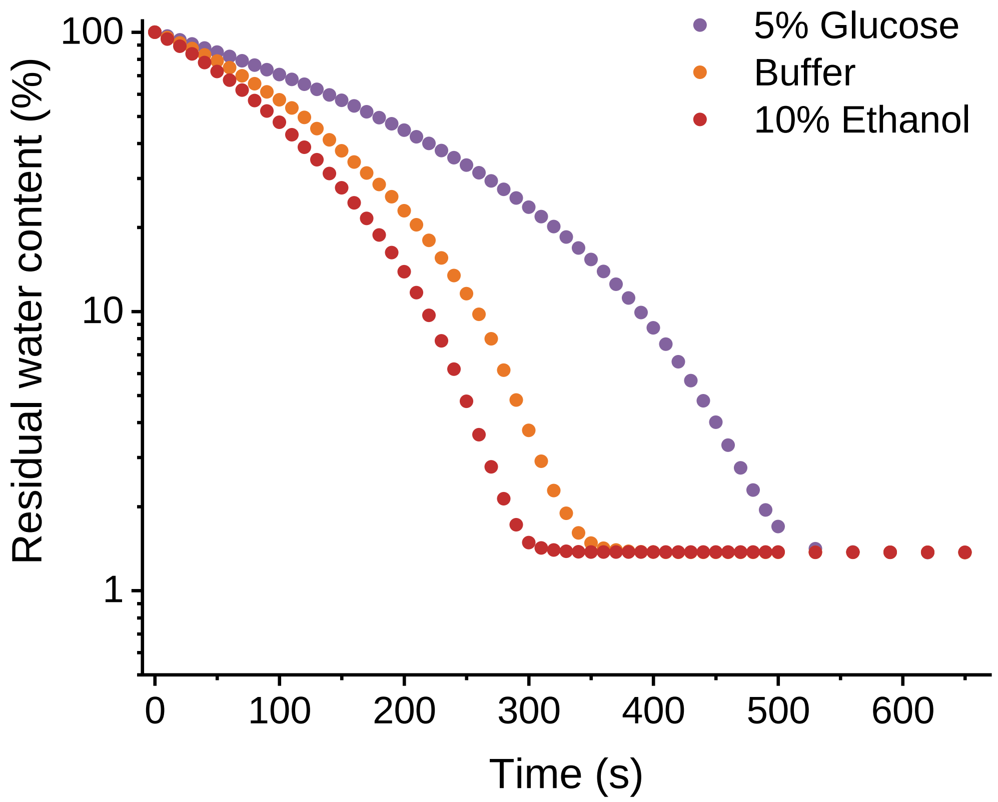

Supplementary Fig. 15. The roughly quantitative description of different evaporation speeds due to different mediums. The residual water content indicates the ratio of water inside porous domain at certain time point vs original water content. Data was obtained by simulation.

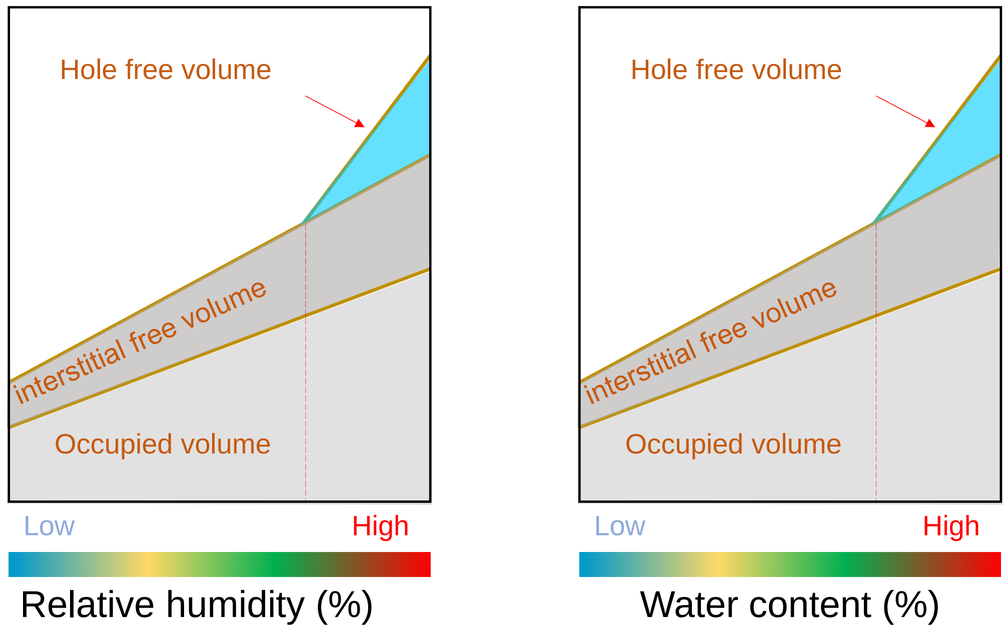

Supplementary Fig. 16. The free volume induced by water sorption in xerogel fibers.

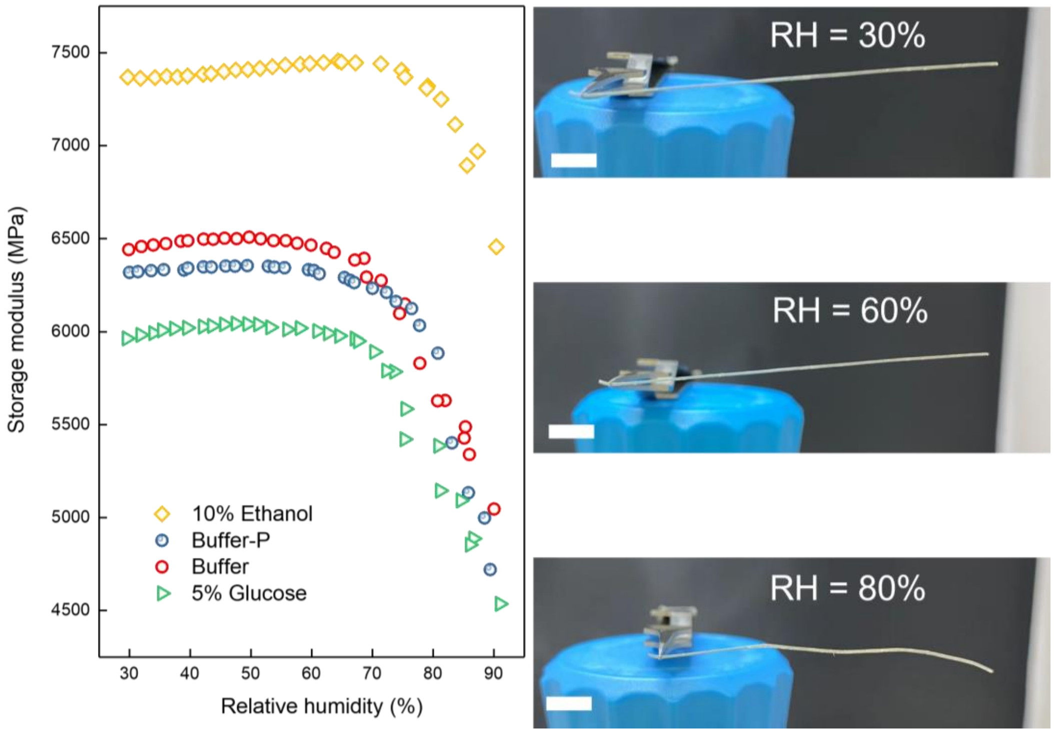

Supplementary Fig. 17. The storage modulus of xerogel fibers using humidity sweep method and the snapshots of xerogel fibers under different relative humidity. Scale bar: 1 cm.

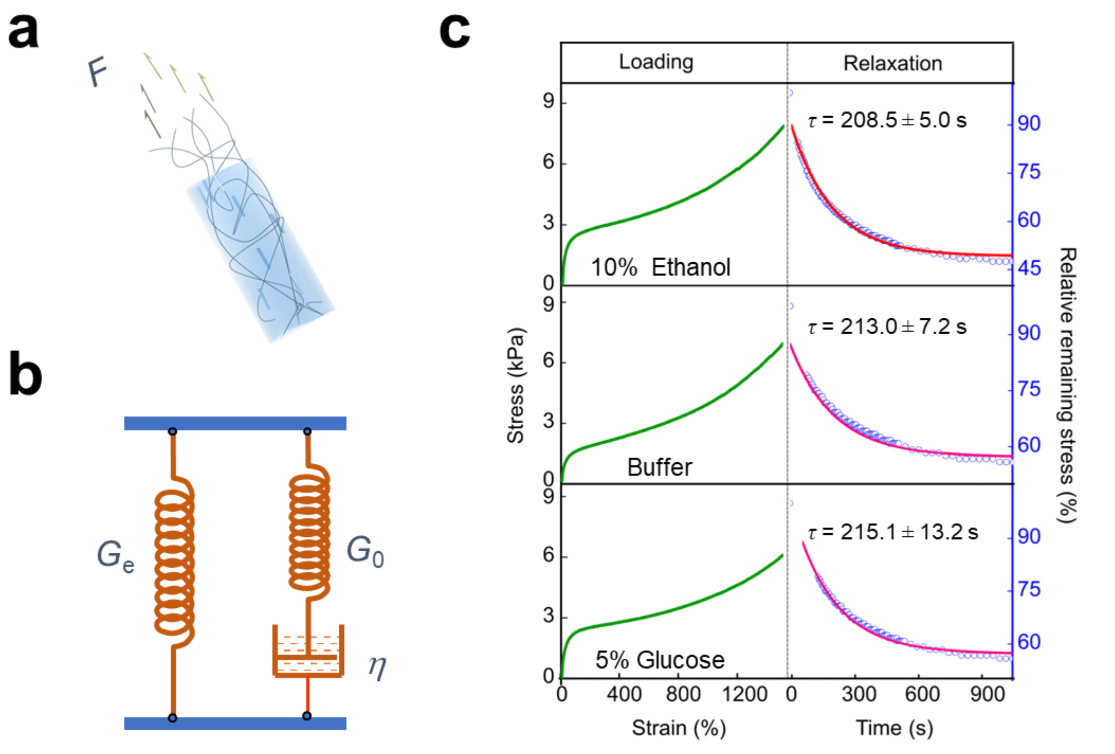

Supplementary Fig. 18 The hydrogel relaxation time physical model. a) The schematic illustration of uniaxial tensile. **b**) A typical Voigt-Kelvin model to describe the stress relaxation process of crosslinked polymer. **c**) The typical stress-relaxation curves of hydrogel fibers (n = 3 independent experiments). Relaxation times are expressed as mean ± SD.

The total stress (G_{all}) is the sum of both parts:

$$G_{all} = G_e + G_0 e^{-\frac{t}{\tau}} \tag{7}$$

Here G_e and G_0 are the stress of spring and dashpot, t and τ are the time after starting relaxation and τ is called relaxation time, which is the important material parameter used to characterize viscoelastic materials. Considering that these three hydrogels were prepared using different solvents, we used the initial stress ($t = 0$) to normalize the stress relaxation which is shown as relative remaining stress. As the initial strain rate applied to establish a constant strain that remains the same for these tests, stress relaxation tests were conducted and demonstrated similar characterizations. All three hydrogels displayed typical viscoelasticity features, in both loading and relaxation process. Using Eq. (7) the stress relaxation process was simulated, the coefficient of determination (R^2) of three samples was higher than 99.5%. Therefore, it was appropriate that we used

Voigt-Kelvin model to describe the actual behavior of CNC hybrid hydrogels in air drying steps.

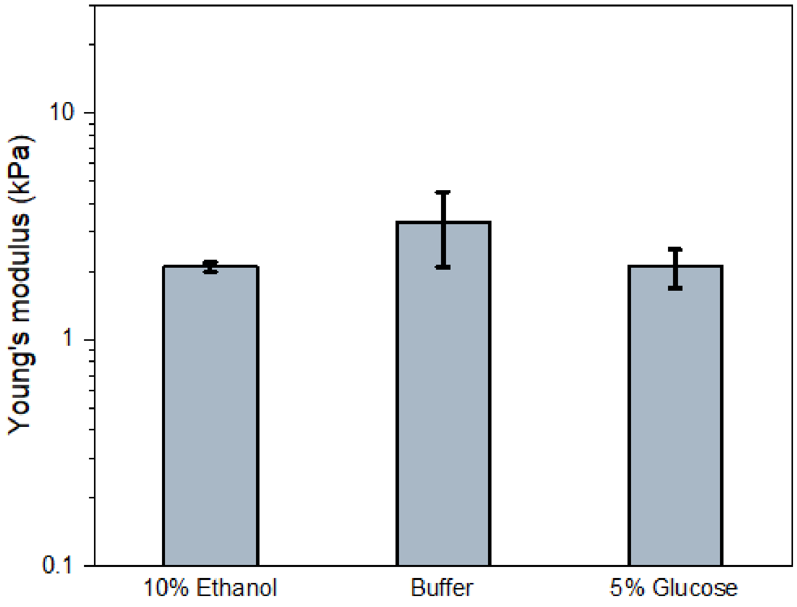

Supplementary Fig. 19. Young's modulus of various hydrogel samples (n = 5 independent experiments). Error bars are SD.

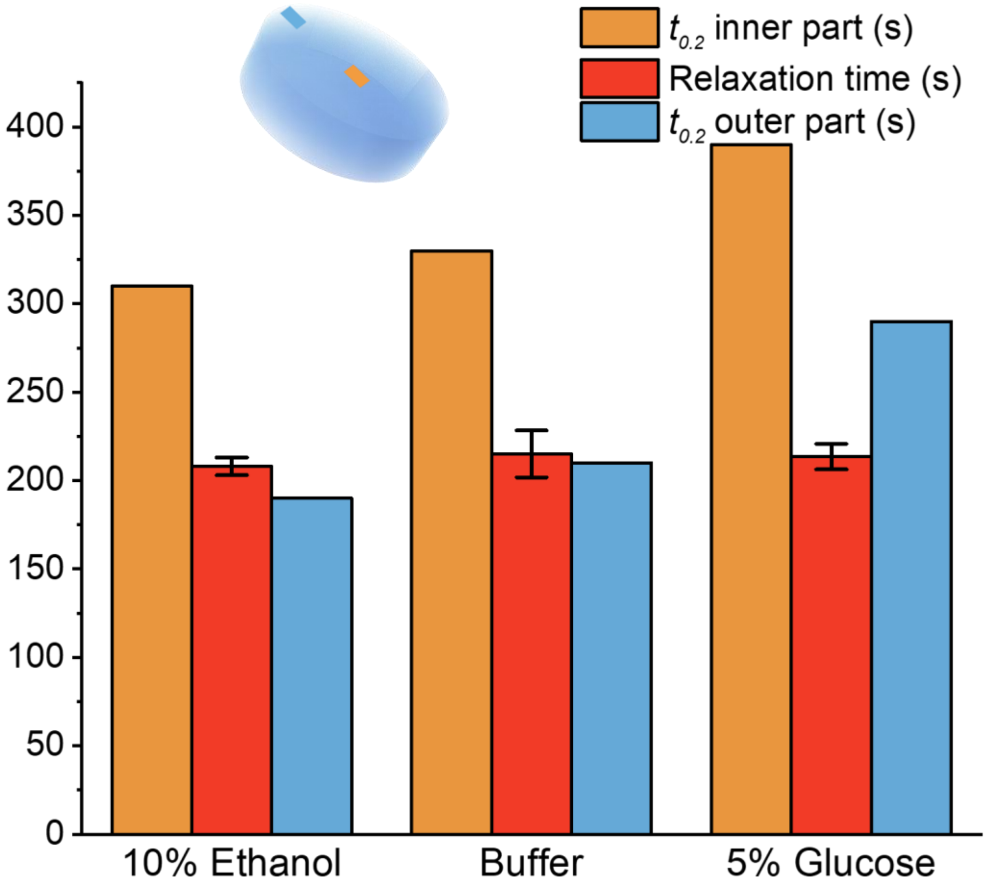

Supplementary Fig. 20. Relaxation times of various hydrogel samples and the time point when the water content was less than 20% for their inner and outer parts (according to the simulation results). n = 3 independent experiments for determine relaxation time. Inset: selected area of inner part and outer part for analysis. Error bars are SD.

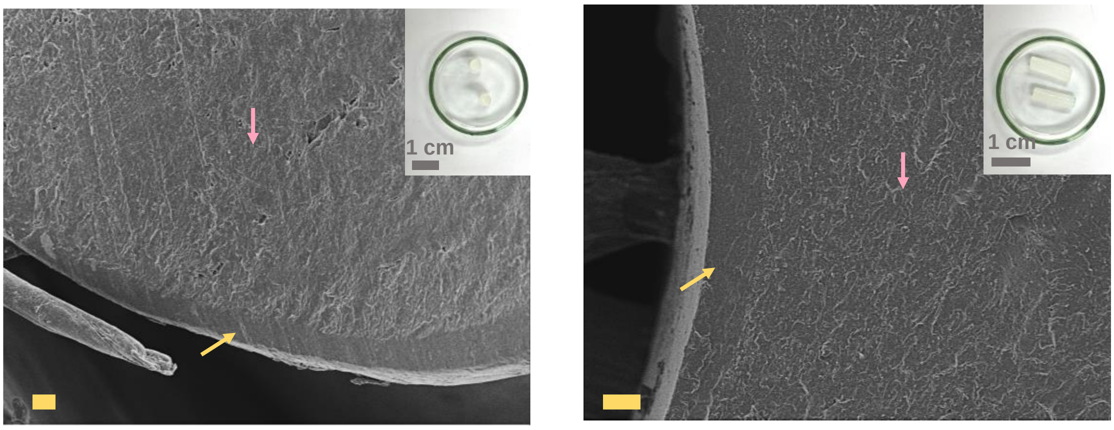

Supplementary Fig. 21. The heterogeneous structures were found in obtained xerogel fibers from buffer samples without stretching. The virgin hydrogels were placed vertically or horizontally. Scale bar: 10 µm. Insets: the original status of hydrogels.

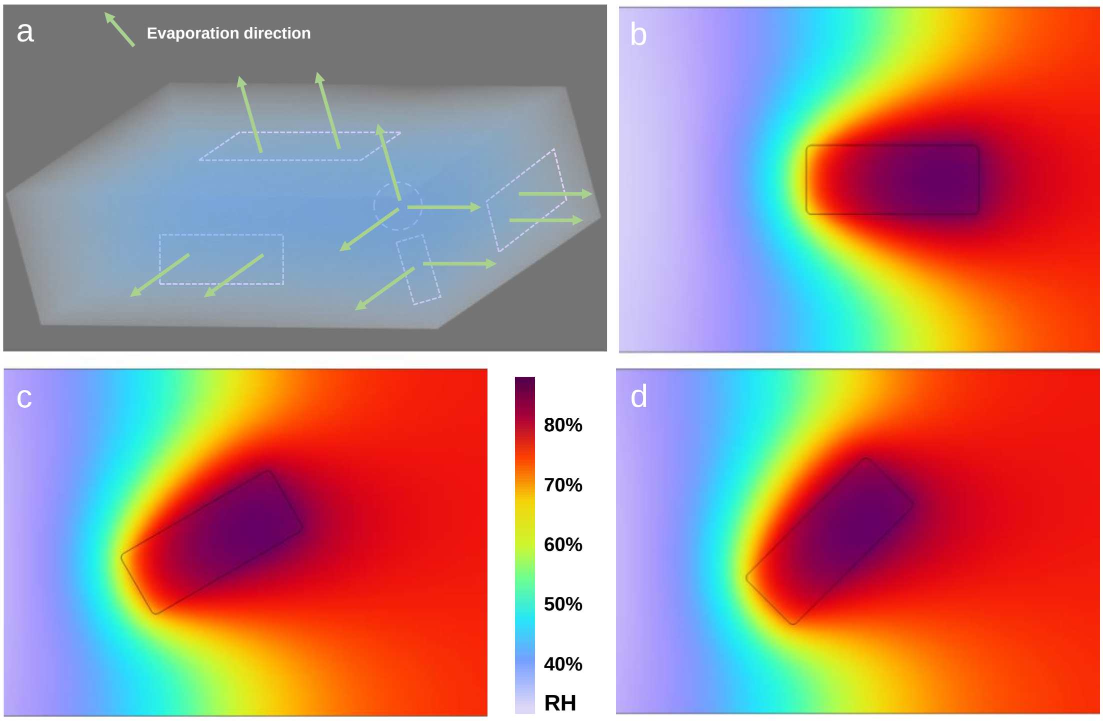

Supplementary Fig. 22. The anisotropic evaporation process for cuboid hydrogels. a). Illustration of the evaporation speed distribution around a cuboid hydrogel sample surface. **b-d**). The relative humidity distribution around the cubic hydrogel sample after 90 s air drying. A cuboid sample was rotated in certain directions to simulate difference in air drying process due to geometric anisotropy (0°, 30°, 45°). Due to anisotropy of the geometric parameters, the evaporation behavior will be intrinsically anisotropy as well.

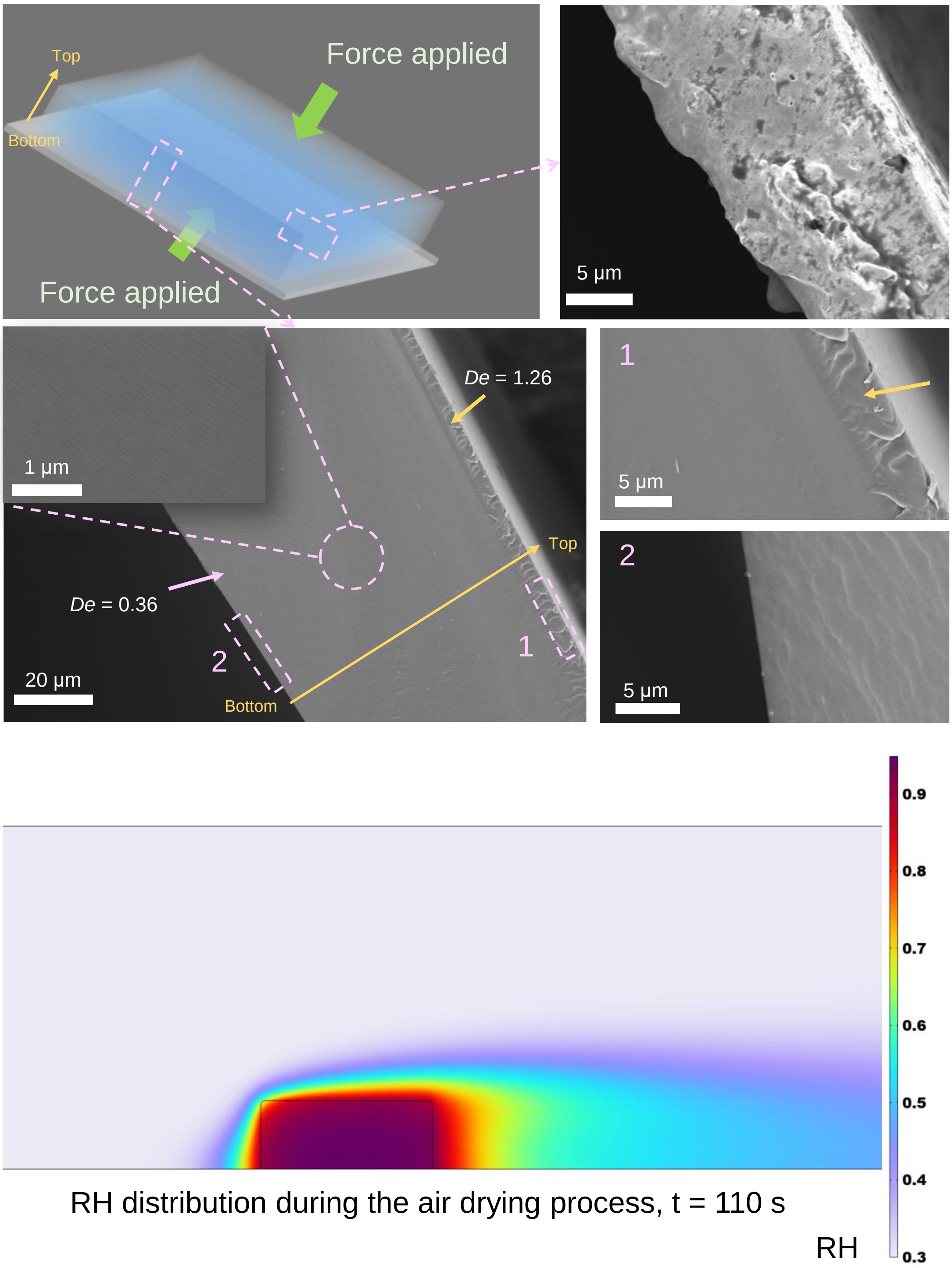

Supplementary Fig. 23. The microstructures of obtained xerogels after applying an external force. The force was applied for about 5 minutes to allow the network reorganization. Then the force was removed. During the evaporation process, one side was placed on the glass and the other side was open. The heterogeneous structure emerged around the corner due to the air drying process, and the heterogeneity was also found in the face region for sufficiently high thickness. This is ascribed to divergent *De*.

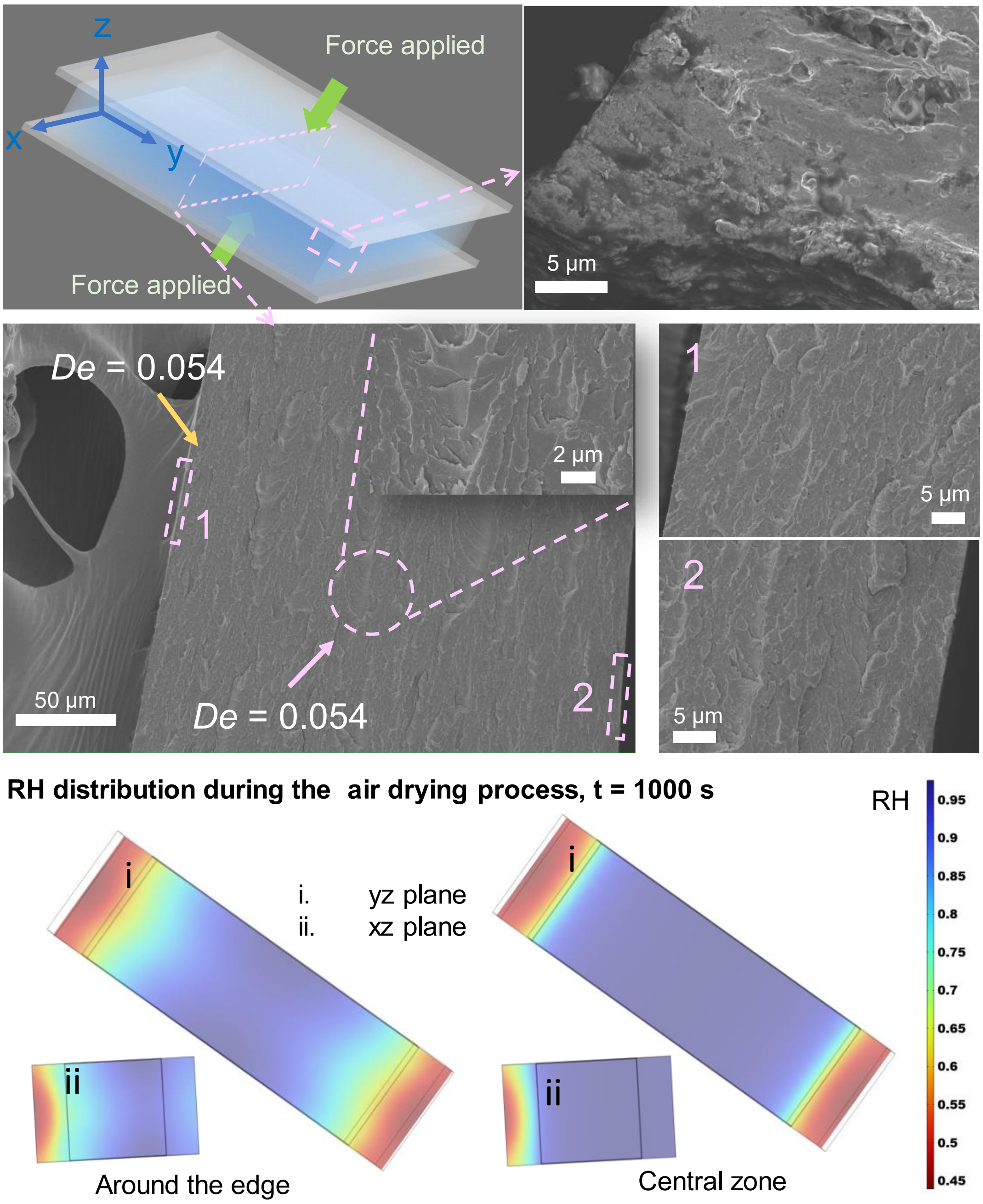

Supplementary Fig. 24. The microstructures of obtained xerogels after applying an external force. The force was removed before air drying. During the evaporation process, both sides were covered with glass slides. The only heterogeneous structure was found around the corner of xerogels. In contrast, the central zone showed the same microstructural features, and no obvious heterogeneity was detected. By numerical simulation it can be found that different evaporation processes induced by the anisotropic geometry are obvious. Dehydration at the edge of the samples is much faster and more anisotropic than in the center. In the center of the samples, the evaporation speed is the same, i.e., those regions are of the same *De* (approx. 0.054, based on a rough model). The same *De* without divergence predicted the homogeneity of the resulting xerogel samples well.

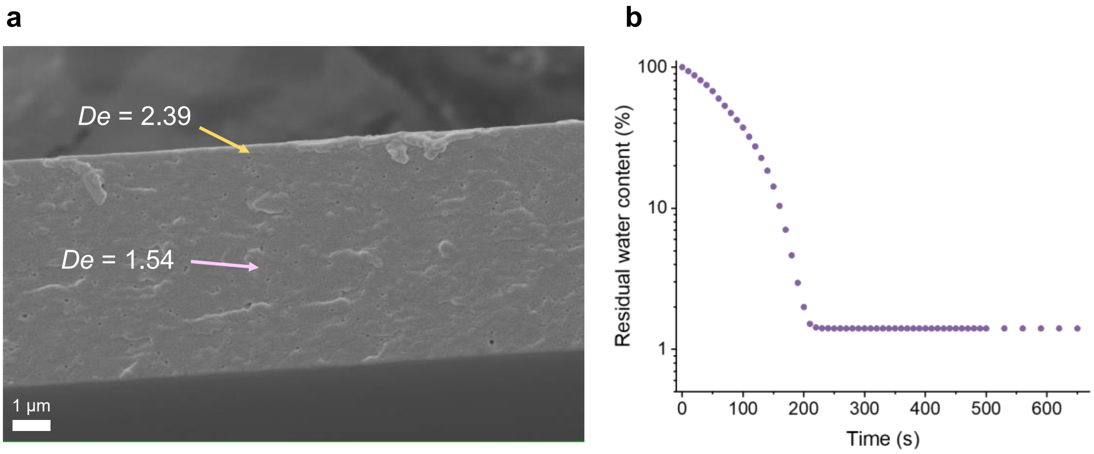

Supplementary Fig. 25. The extremely thin xerogel obtained by using dynamically crosslinked hydrogels after air drying. **a**) The microstructure of xerogel films; **b**) The residual water content in air drying process. Due to the rather small thickness and the large evaporation area, the *De* for both the boundary and the central region exceed 1 without divergence and as expected the heterogeneous structures are not detected.

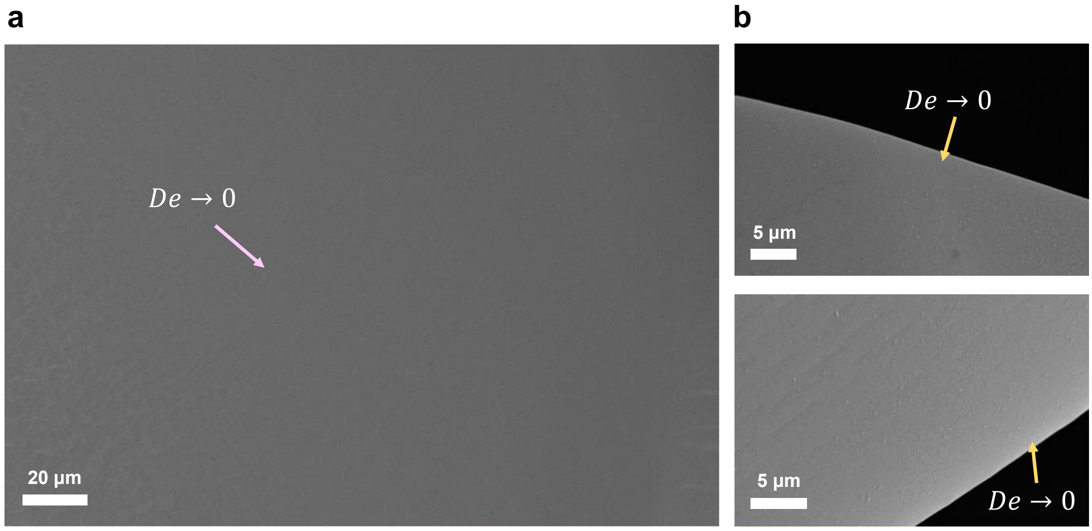

Supplementary Fig. 26. The microstructures of the xerogels obtained from covalently crosslinked hydrogels, a cylindrical hydrogel was applied before air drying. a) Central region, **b**) Boundary region. The relaxation time of such covalently crosslinked hydrogel is in the order of 10^{-5} s, which is assigned to the concentration fluctuations of the polymer chains in the covalent crosslinked hydrogels[1,2]. Thus, the *De* for central or boundary region was approached to zero. Both the boundary zone and the central zone showed almost the same structural characteristics according to the SEM images.

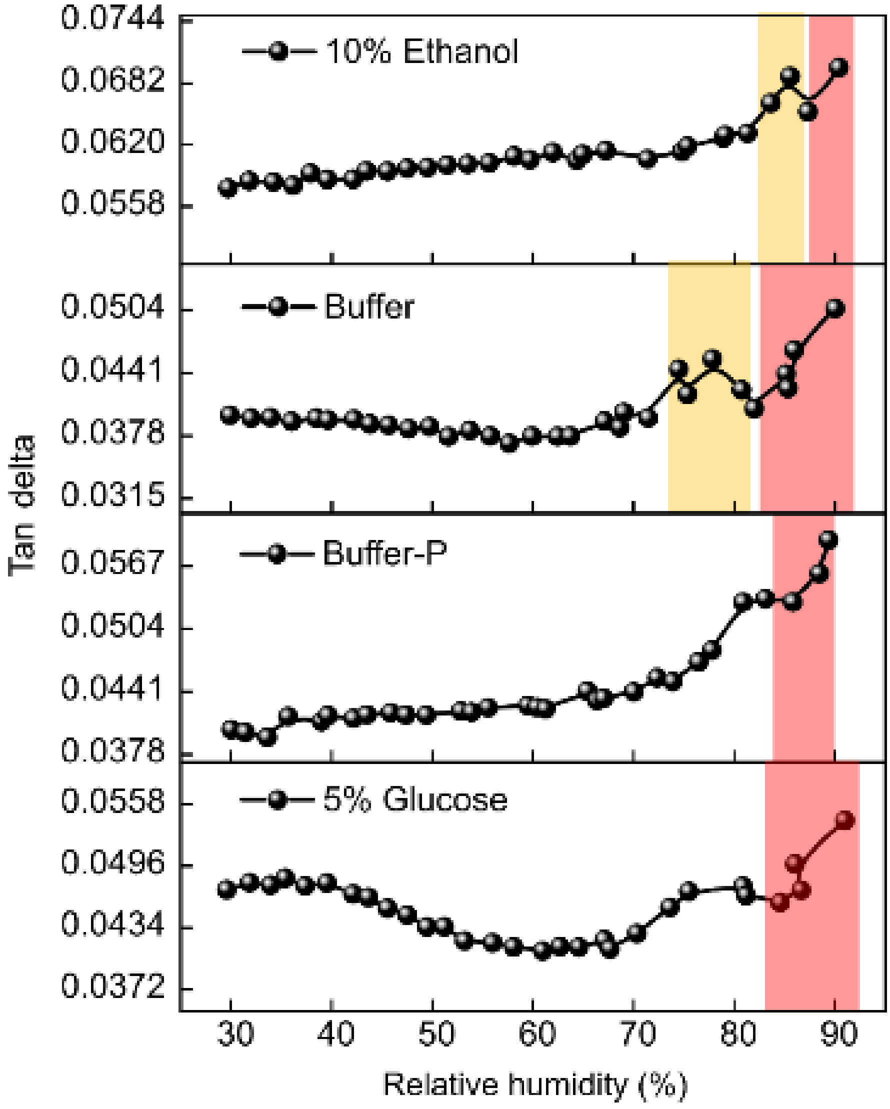

Supplementary Fig. 27. Loss factors of four different xerogel fibers as measured from humidity sweep tests on DMTA. The second order transition (brown region) was found in both 10% ethanol and buffer fibers, which were induced by the existence of an outer layer.

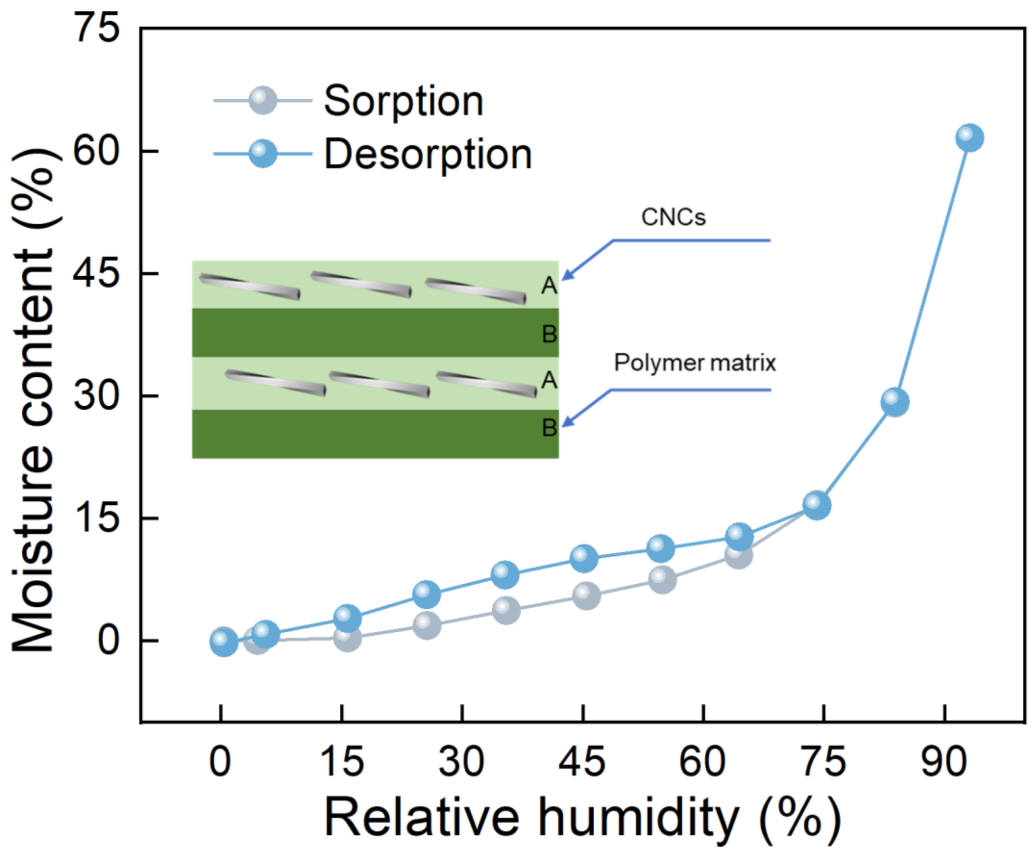

Supplementary Fig. 28. The typical isothermal sorption-desorption curves of xerogel fibers. The type B hysteresis loops are associated with slit shaped pores.

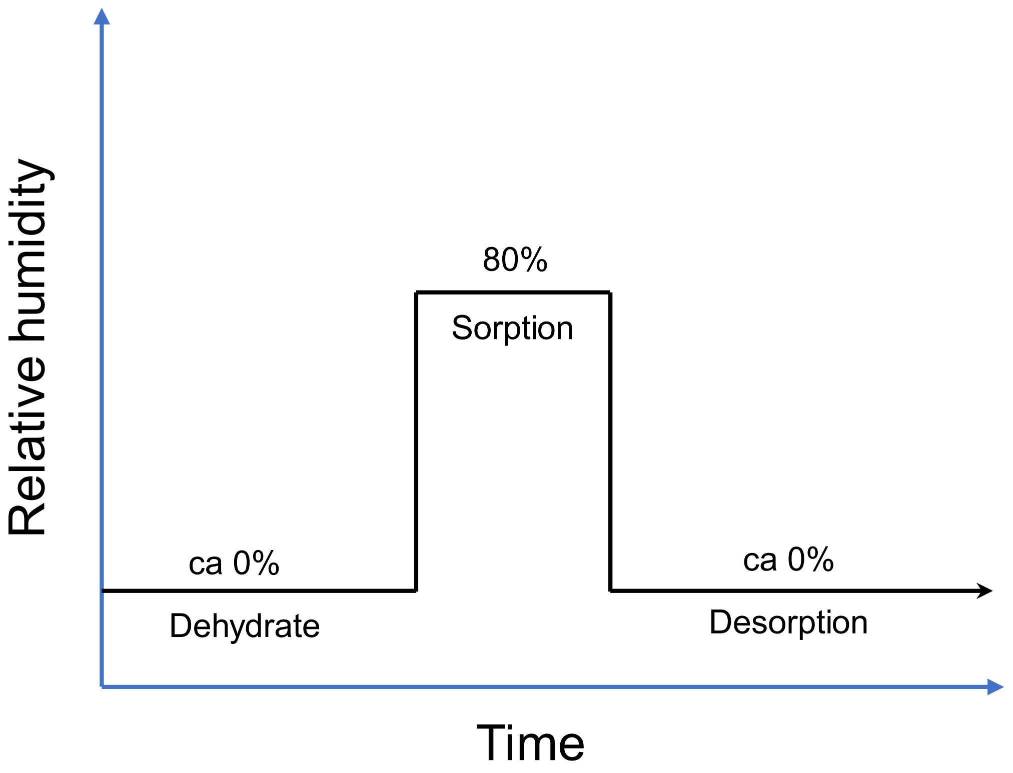

Supplementary Fig. 29. Method to determine the sorption-desorption properties of xerogel fiber under consistent temperature.

Supplementary Table 2. Aspect ratio of different samples

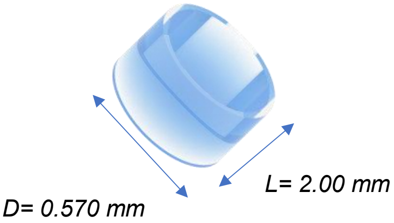	L/D = 3.5
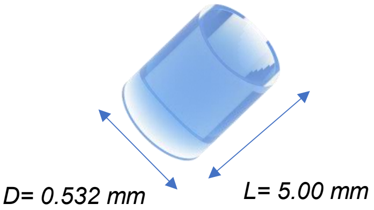	L/D = 9.4
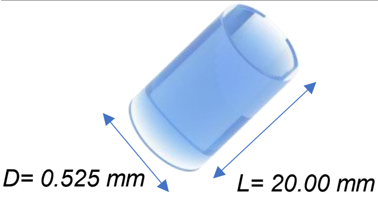	L/D = 38.1

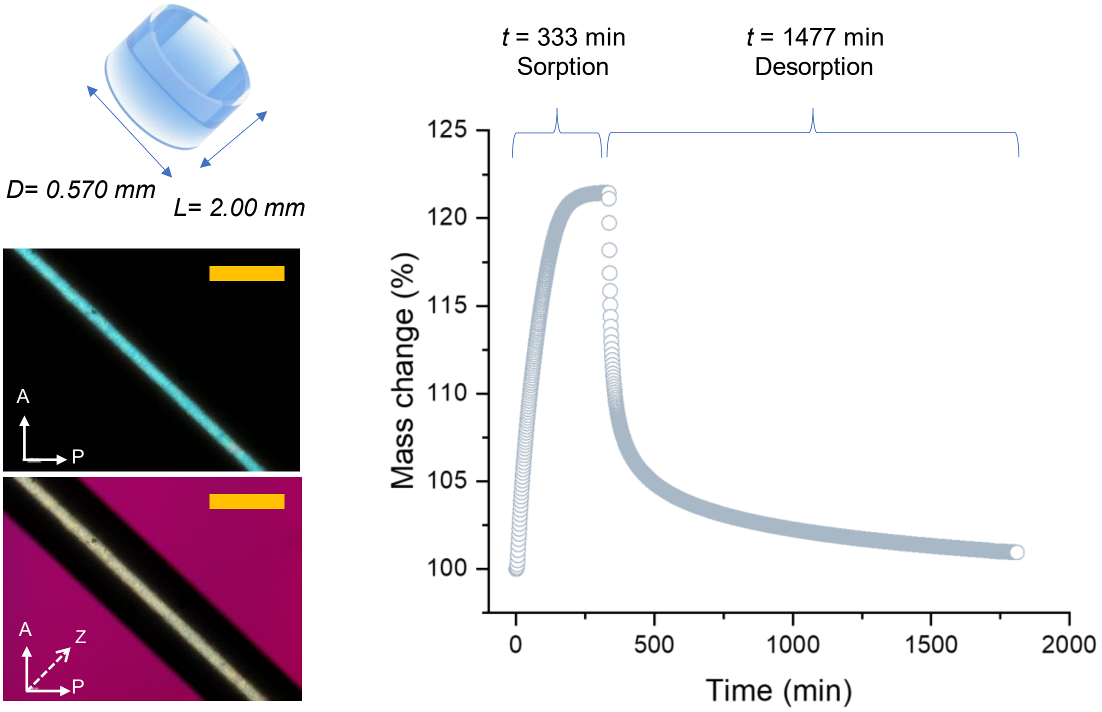

Supplementary Fig. 30. Sorption-desorption behavior of xerogel fiber (formed in buffer, length 2.00 mm). The geometric shape and optical properties of xerogel and typical water vapor sorption-desorption curve of xerogel were shown. Scale bar: 500 µm.

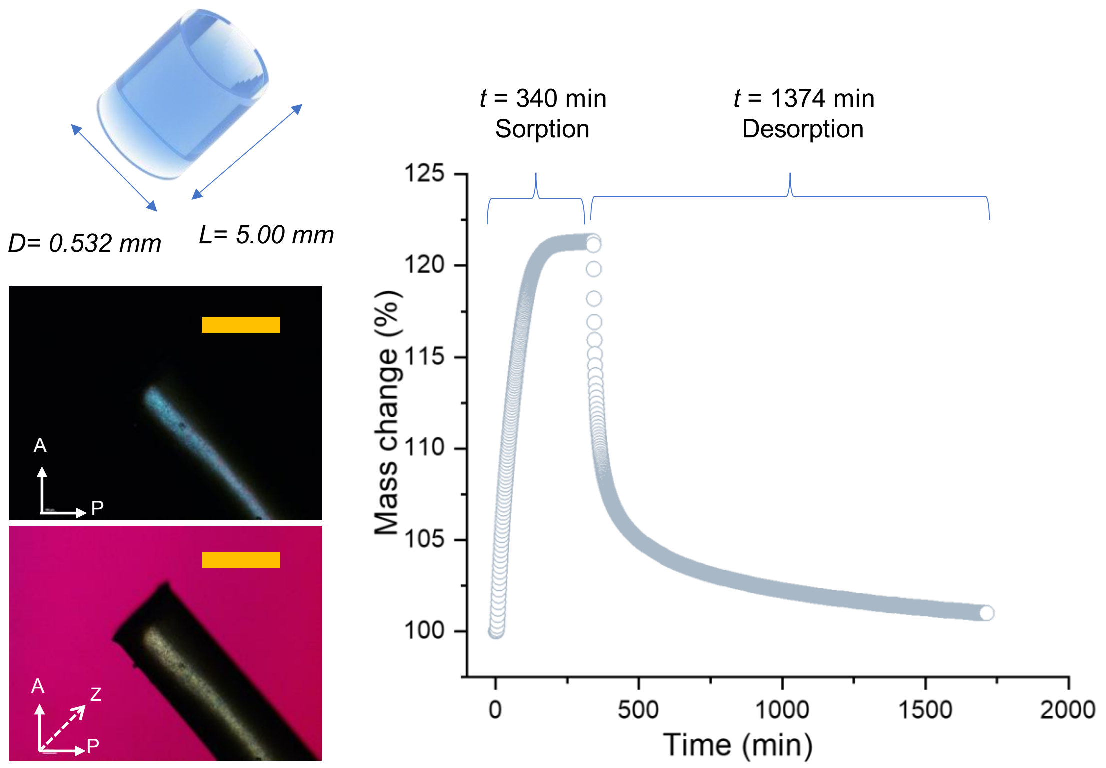

Supplementary Fig. 31. Sorption-desorption behavior of xerogel fiber (formed in buffer, length 5.00 mm). The geometric shape and optical properties of xerogel and typical water vapor sorption-desorption curve of xerogel were shown. Scale bar: 500 µm.

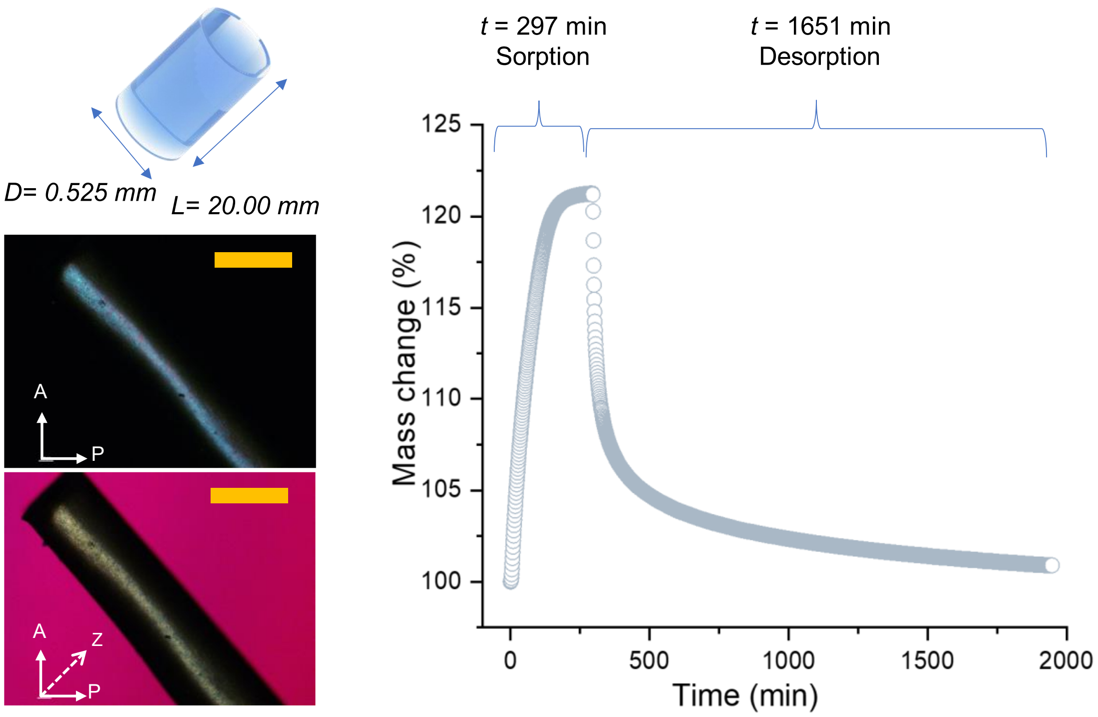

Supplementary Fig. 32. Sorption-desorption behavior of xerogel fiber (formed in buffer, length 20.00 mm). The geometric shape and optical properties of xerogel and typical water vapor sorption-desorption curve of xerogel were shown. Scale bar: 500 µm.

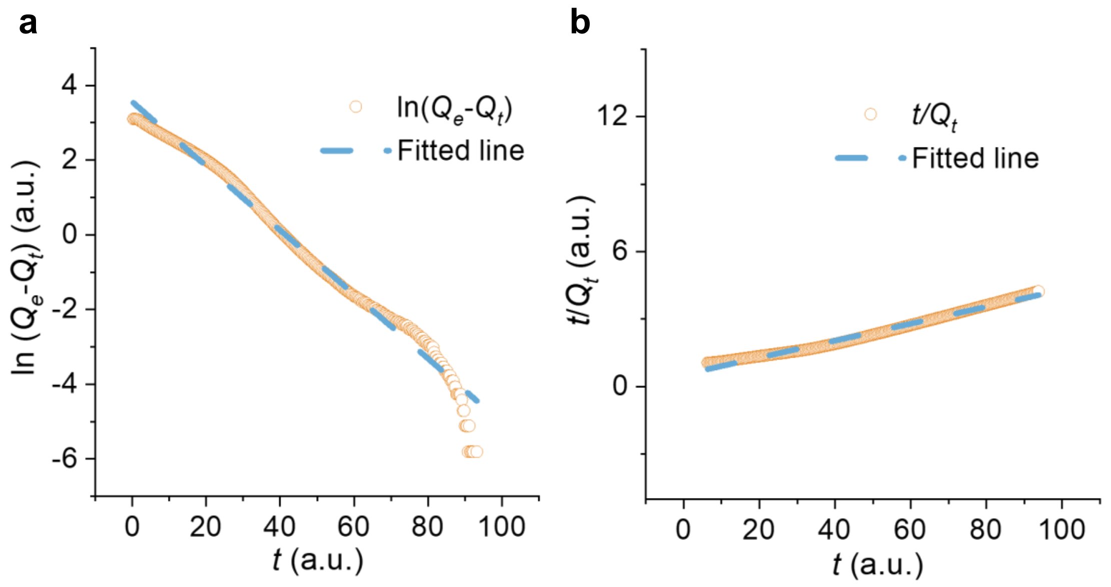

Supplementary Fig. 33. The fitting line for the sorption curve. **a)** Pseudo-first-order and **b)** Pseudo-second-order adsorption kinetic fitting curves of the typical sorption curves.

Supplementary Table 3. Kinetic parameters for sorption of water vapor by hybrid xerogel fibers

Sample		1	2	3
Aspect ratio		3.5	9.4	38.1
Experimental sorption capacity		21.43	21.24	21.32
Pseudo 1st-order sorption kinetics	Q_e	32.52	24.43	24.48
	K_1	0.1178	0.0739	0.0627
	R^2	0.9638	0.9873	0.9933
Pseudo 2nd-order sorption kinetics	Q_e	28.36	27.93	25.37
	K_2	0.0021	0.0019	0.002
	R^2	0.9837	0.9824	0.9874
Weber and Morris intraparticle diffusion	K_{id1}	0.1626	0.2595	0.0646
	K_{id2}	3.9411	3.5212	3.0028
	K_{id3}	0.2157	0.1679	0.0839

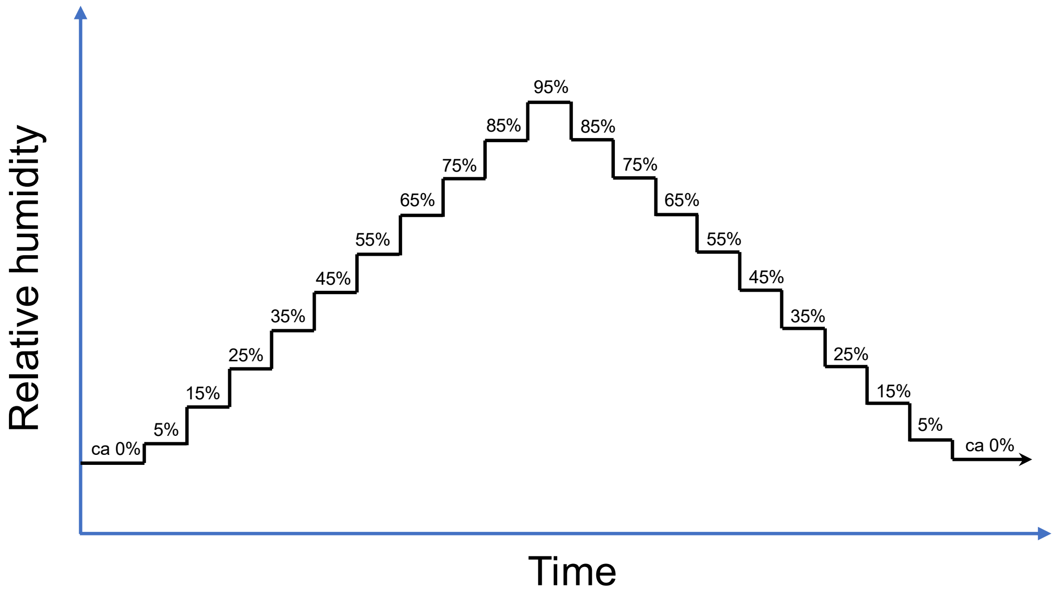

Supplementary Fig. 34. A typical sorption-desorption method to determine the sorption isotherm of xerogel fibers.

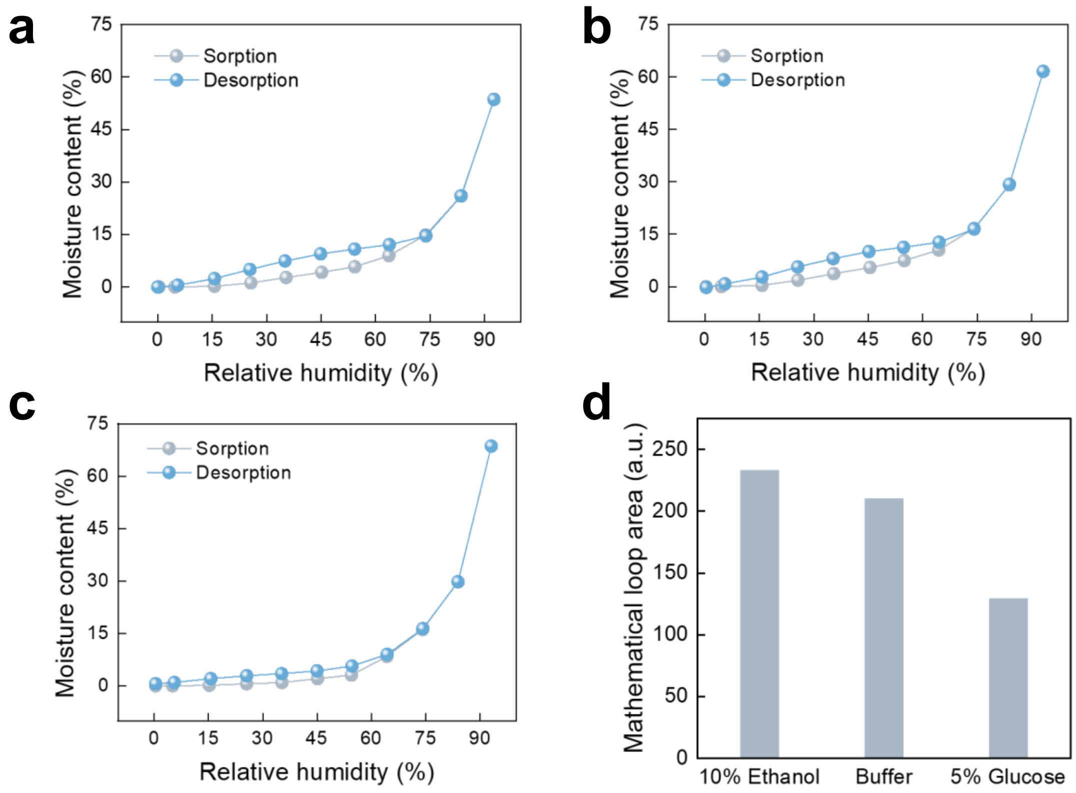

Supplementary Fig. 35. Sorption and desorption isotherms of xerogel fibers. a) 10% ethanol. **b)** Buffer. **c)** 5% glucose. **d)** The mathematic area of hysteresis loop of various samples.

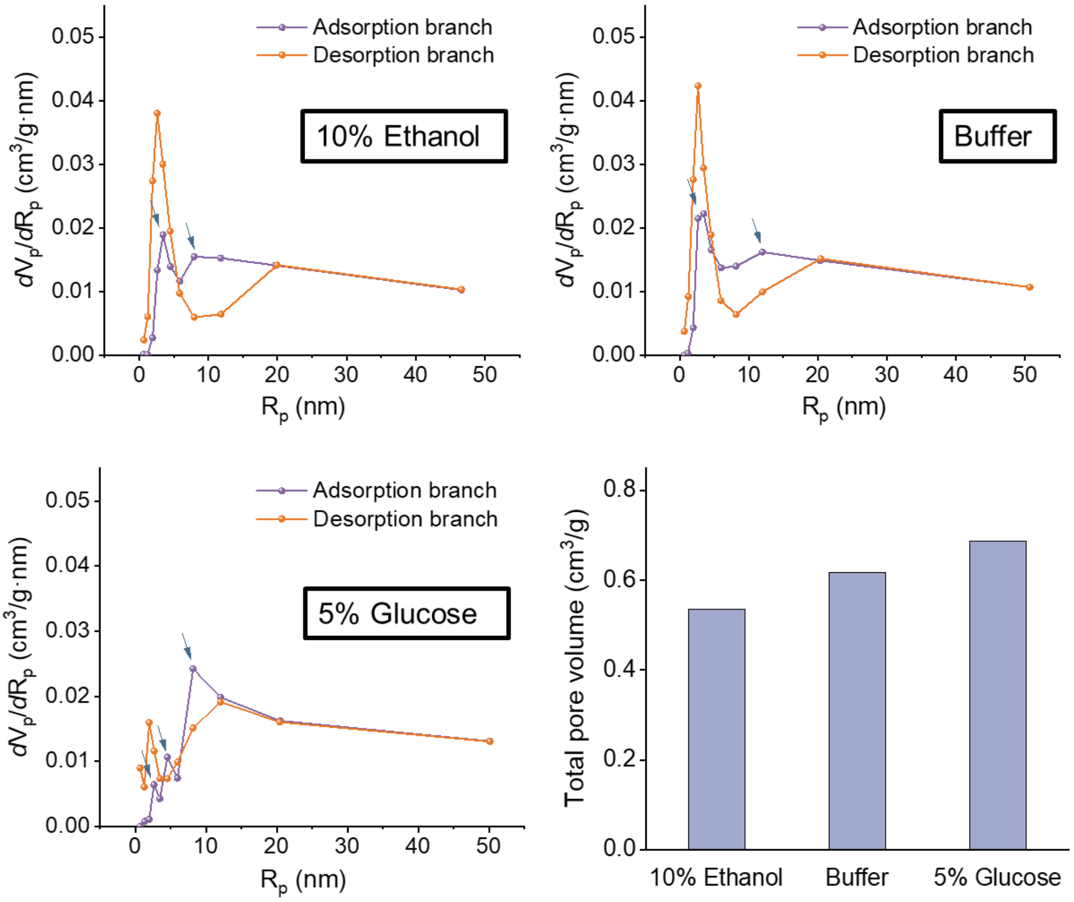

Supplementary Fig. 36. BJH Pore Size Distribution (PSD) of samples and pore size peak positions for various xerogel samples. The total pore volume was calculated at a P/P_0 of 0.93.

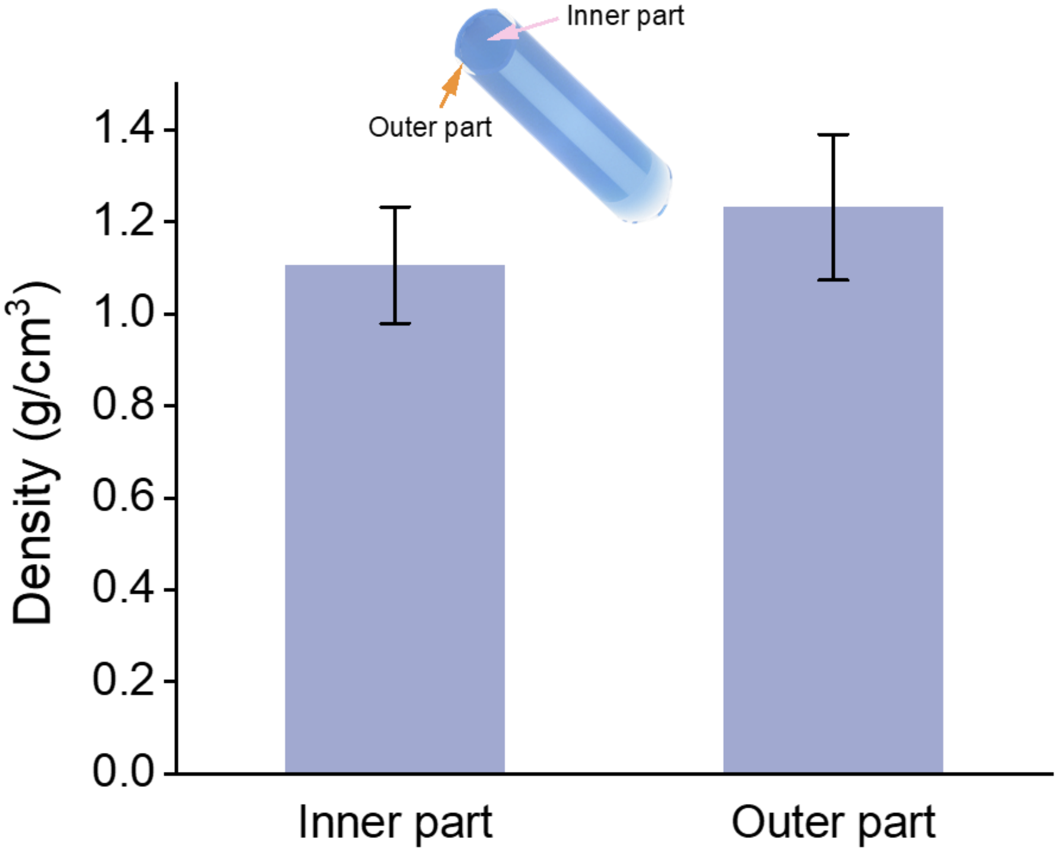

Supplementary Fig. 37. Densities of different parts in the xerogel fibers. The buffer samples were taken as examples, assuming that the samples were perfect cylinders with only two different parts inside. The outer layer was removed mechanically. By recording the mass and diameter of the xerogel samples, the densities of different parts were calculated (n = 5 independent experiments). Error bars are SD.

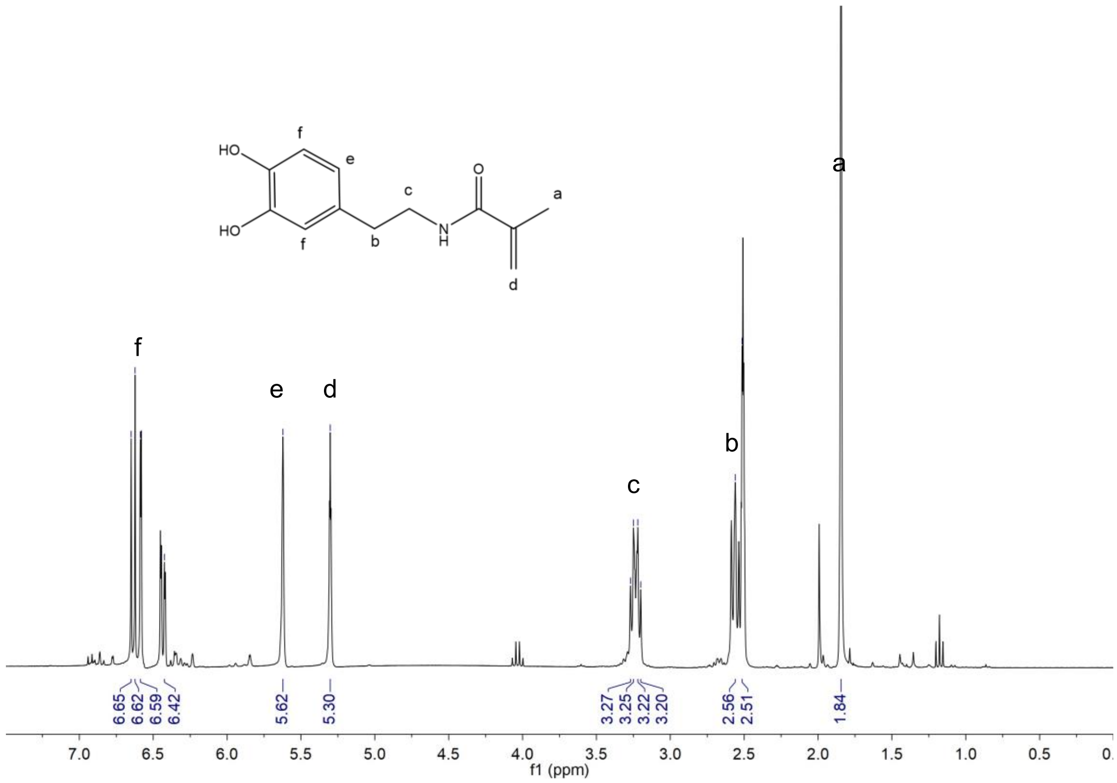

Supplementary Fig. 38. ^{1}H-NMR of DMA with peak assignments (400 MHz, DMSO-*d*, 298 K).

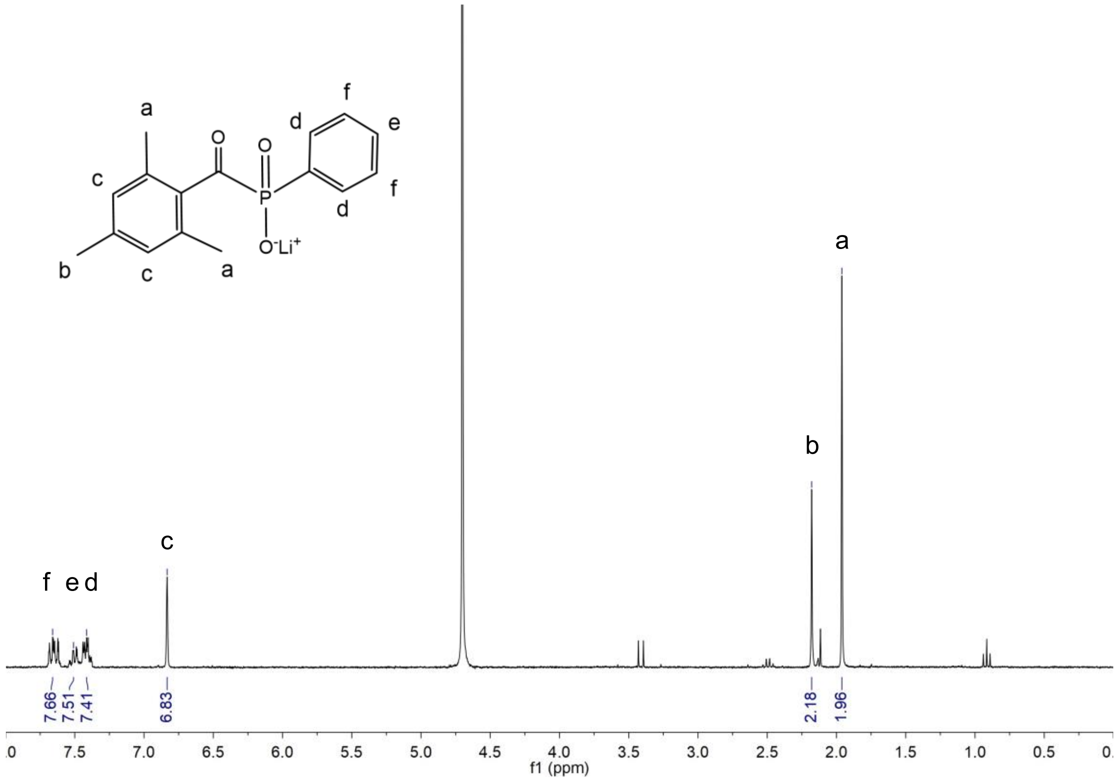

Supplementary Fig. 39. ^{1}H-NMR of LAP with peak assignments (400 MHz, D_2O, 298 K).

Supplementary References:

1. Cao Z*, et al.* Tough, ultrastretchable and tear-resistant hydrogels enabled by linear macro-cross-linker. *Polym. Chem.* **10**, 3503-3513 (2019).

2. Huang M, Furukawa H, Tanaka Y, Nakajima T, Osada Y, Gong JP. Importance of entanglement between first and second components in high-strength double network gels. *Macromolecules* **40**, 6658-6664 (2007).
