# Supplementary material for: Divergent Deborah number-dependent transition from homogeneity to heterogeneity
Source: Nat Commun. 2023 Sep 26;14:6003. doi: 10.1038/s41467-023-41738-0 (PMC10522598; doi:10.1038/s41467-023-41738-0)
Supplement: Supplementary file 3 — Description of Additional Supplementary Files [file 41467_2023_41738_MOESM3_ESM.pdf]

Description of Additional Supplementary Files

Supplementary Movie 1. The evolution of relative humidity in hydrogel fiber in the stage of air drying. The time changes from 0 to 1000 s.
